# Lead‐Free Halide Perovskites for Light Emission: Recent Advances and Perspectives

**DOI:** 10.1002/advs.202003334

**Published:** 2021-01-04

**Authors:** Xin Li, Xupeng Gao, Xiangtong Zhang, Xinyu Shen, Min Lu, Jinlei Wu, Zhifeng Shi, Vicki L. Colvin, Junhua Hu, Xue Bai, William W. Yu, Yu Zhang

**Affiliations:** ^1^ State Key Laboratory of Integrated Optoelectronics and College of Electronic Science and Engineering Jilin University Changchun 130012 China; ^2^ Key Laboratory for Special Functional Materials of Ministry of Education National & Local Joint Engineering Research Centre for High‐Efficiency Display and Lighting Technology School of Materials and Engineering Collaborative Innovation Centre of Nano Functional Materials and Applications Henan University Kaifeng 475000 China; ^3^ Key Laboratory of Materials Physics of Ministry of Education Department of Physics and Engineering Zhengzhou University Zhengzhou 450052 China; ^4^ Department of Chemistry Brown University Providence RI 02912 USA; ^5^ State Centre for International Cooperation on Designer Low‐carbon & Environmental Materials School of Materials Science and Engineering Zhengzhou University Zhengzhou 450001 China; ^6^ Department of Chemistry and Physics Louisiana State University Shreveport LA 71115 USA

**Keywords:** lead‐free perovskites, LEDs, light emission, nanomaterials

## Abstract

Lead‐based halide perovskites have received great attention in light‐emitting applications due to their excellent properties, including high photoluminescence quantum yield (PLQY), tunable emission wavelength, and facile solution preparation. In spite of excellent characteristics, the presence of toxic element lead directly obstructs their further commercial development. Hence, exploiting lead‐free halide perovskite materials with superior properties is urgent and necessary. In this review, the deep‐seated reasons that benefit light emission for halide perovskites, which help to develop lead‐free halide perovskites with excellent performance, are first emphasized. Recent advances in lead‐free halide perovskite materials (single crystals, thin films, and nanocrystals with different dimensionalities) from synthesis, crystal structures, optical and optoelectronic properties to applications are then systematically summarized. In particular, phosphor‐converted LEDs and electroluminescent LEDs using lead‐free halide perovskites are fully examined. Ultimately, based on current development of lead‐free halide perovskites, the future directions of lead‐free halide perovskites in terms of materials and light‐emitting devices are discussed.

## Graphical abstract

The fundamental reasons for efficient emission of halide perovskites are investigated, which is helpful to get efficient light emission of lead‐free halide perovskites. The synthesis, crystal structure, optical and optoelectronic properties of different molecular dimensional lead‐free halide perovskites with different forms are then systematically reviewed. Finally, the applications of light‐emitting devices (phosphor‐converted LEDs and electroluminescent LEDs) are discussed.

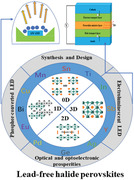

## 1 Introduction

Recently, lead halide perovskite materials have attracted increasing attention since the reported power conversion efficiency (PCE) of solar cells based on them soared from an initial value of 3.8% in 2009 to 25.2% in 2020.^[^ ^1^ ^]^ The high PCE originates from exceptional photovoltaic properties of perovskites, including high absorption of light, small exciton binding energy, balanced electron and hole mobilities, long exciton diffuse length, and low defect density.^[^ ^2^ ^]^ Xiao et al. studies that the underlying reasons in detail, including the electronic configuration of Pb, high electronic dimensionality, and high perovskite symmetry (O_h_) for these outstanding properties.^[^ ^3^ ^]^ The high PCE motivated the researchers to study LEDs. As expected, lead halide perovskites have also been confirmed to be an excellent light emitter in recent years, which have been widely studied in light‐emitting devices, owing to high PLQY, narrow peak full width at half maximum (FWHM), tunable spectral range, and facile synthesis.^[^ ^4^ ^]^ Over a span of five years, an ocean of investigations related to lead halide perovskites have been carried out, including doping, surface passivation, surface coating, and postsynthetic treatment.^[^ ^4^, ^5^ ^]^ Moreover, these studies have also made great strides in concomitant with outstanding performance.^[^ ^6^ ^]^

Currently, organic LEDs (OLEDs) and quantum dot LEDs (QLEDs) have been commercialized in display technology, such as OLED mobile phone from Huawei, OLED television from LG, QLED television from Samsung.^[^ ^7^ ^]^ In addition to displays, OLEDs and QLEDs also show potentials for lighting applications. However, OLEDs and QLEDs present some inherent disadvantages. OLEDs possess lower color purity and narrower color gamut. Additionally, OLEDs are fabricated through vacuum‐based thermal evaporation, which is not suitable for cost‐effective mass production.^[^ ^8^ ^]^ The quantum dots used in QLEDs provide better color purity, excellent stability, and high efficiency, but they (especially the core‐multiple–shell structures) require extremely complex high‐temperature synthesis and expensive raw materials.^[^ ^9^ ^]^ The comparisons of perovskites, quantum dots, and organic fluorophores for photoluminescence (PL), FWHM, PLQY and color gamut are listed in **Table** **1**.

**Table 1:** Photoluminescence comparison of perovskites, conventional quantum dots, and organic fluorophores

Parameter	CsPbX_3_ (X = Cl, Br, I)	Conventional quantum dot	Organic fluorophore
PL (nm)	410–700	450–650	450–650
FWHM (nm)	15–35	25–40	>45
PLQY (%)	50–100	50–98	50–90
Color gamut (%)	≈140	≈120	≈90

In addition to the superior optical properties, the synthesis process of perovskites is quite simple at room or relatively low temperature (<180 °C).^[^ ^10^ ^]^ More importantly, perovskites possess balanced carrier mobility and long exciton diffusion length, which are beneficial to LEDs.^[^ ^11^ ^]^ Owing to the above excellent properties, lead halide perovskite light‐emitting devices have obtained superior external quantum efficiencies (EQEs) exceeding 20% in recent years.^[^ ^12^ ^]^

Lead halide perovskites have superior properties for light emission, however, the existence of lead element restricts their mass applications. It is noteworthy that one of the degradation products of lead halide perovskites embraces water‐soluble Pb salt.^[^ ^13^ ^]^ A great deal of medical research has reported the severe toxicity of lead. Low‐level lead exposures can disturb the neurological, cardiovascular, and hematopoietic systems.^[^ ^14^ ^]^ The Centers for Disease Control and Prevention (CDC) of the United States recommends <10 µg per deciliter blood lead levels to be safe.^[^ ^15^ ^]^ In 2003, the European Union released “Directive on the restriction of the use of certain hazardous substances containing lead in electrical and electronic equipment,” which demanded lead‐free in electrical and electronic equipment. Thus, developing environmentally benign lead‐free halide perovskites and perovskite analogue materials with excellent optical and optoelectronic properties is urgent and essential.

**Figure 1:**
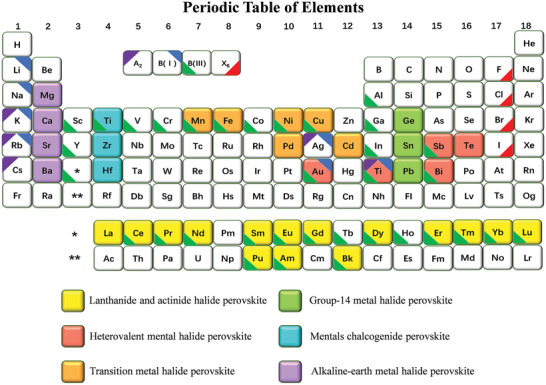
Lead replacement candidates in halide perovskites in the periodic table of elements. Reproduced with permission.^[^ ^17^ ^]^ Copyright 2017, Springer. Elements forming halide double perovskites with the formula A_2_B(I)B(III)X_6_. Reproduced with permission.^[^ ^18^ ^]^ Copyright 2016, American Chemical Society.

Since CH_3_NH_3_SnI_3_ was first introduced by Snaith's team to serve as a photoactive material, a multitude of lead‐free halide perovskite materials have been reported, which are widely applied in solar cells, photodetectors, LEDs, and field‐effect transistors (FETs).^[^ ^16^ ^]^ **Figure** **1** lists the possible replacement of lead in perovskites and perovskite analogues. In fact, some lead‐free perovskites should be known as perovskite analogues from the strict definition of perovskites that are confined in 3D connected BX_6_ octahedra (the crystal cells). Herein, we term the perovskite analogues as perovskites for convenience, aligned with the common definition.

In this review, we introduce the deep‐seated reasons for halide perovskites with excellent optical properties, and discuss the relatively inferior luminescent performances of lead‐free halide perovskites, which can give some foundations for the development of the lead‐free halide perovskites. In addition, the design and synthesis methods of lead‐free halide perovskites are systematically summarized. Subsequently, a comprehensive review on optoelectronic characteristics of lead‐free halide perovskites with different molecular dimensionalities is provided, including single crystals, nanocrystals, and thin films. Their light‐emitting applications are also fully delved. Finally, we put forward some insightful considerations toward future development of lead‐free perovskites and their LEDs.

## 2 Excellent Properties of Lead Halide Perovskites for Light Emission

In order to understand lead‐free halide perovskites, it is essential to know their exceptional properties for light emission. The defect tolerance, bandgap type, and exciton binding energy are several key factors.

### 2.1 Defect Tolerance

Defect tolerance exists in lead halide perovskite thin films and nanocrystals.^[^ ^19^ ^]^ Taking nanoscale materials as an example, defects cannot be ignored due to a high surface to volume ratio.^[^ ^20^ ^]^ In most cases, defects serve as an adverse role and need to be eliminated to the maximum extent. In order to illustrate the importance of defect tolerance, we compare the defect‐intolerant conventional semiconductor with defect‐tolerant lead halide perovskites.^[^ ^21^ ^]^

**Figure 2:**
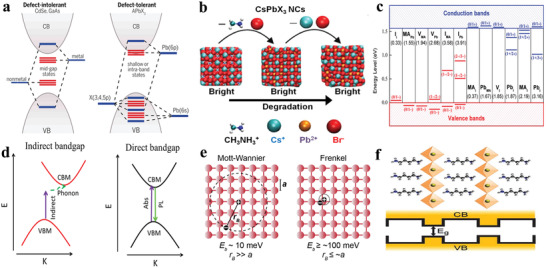
a) Electronic band structure of conventional semiconductor (for example, CdSe, GaAs) and lead halide perovskites. Reproduced with permission.^[^ ^22^ ^]^ Copyright 2017, American Chemical Society. b) Schematic representation that CsPbBr_3_ still keeps bright emission even with the removal of the surface atoms and ligands. Reproduced with permission.^[^ ^23^ ^]^ Copyright 2016, American Chemical Society. c) calculated transition energy of point defects in CH_3_NH_3_PbI_3_. The values in the parentheses represent the formation energies of neutral defects. MA (CH_3_NH_3_) is indicated. Reproduced with permission.^[^ ^2b^ ^]^ Copyright 2017, American Chemical Society. d) Absorption and recombination processes between indirect bandgap and direct bandgap of semiconductor. Reproduced with permission.^[^ ^24^ ^]^ Copyright 2018, American Chemical Society. e) Mott‐Wannier excitons and Frenkel excitons in an arbitrary atomic lattice. The lattice constant *a* and Bohr radius *r*_B_ are indicated. Reproduced with permission.^[^ ^25^ ^]^ Copyright 2016, Royal Society of Chemistry. f) Schematic representation of the natural quantum‐well structures, wherein the inorganic layers act as “wells” and the organic molecules as “barriers.” Reproduced with permission.^[^ ^11^ ^]^ Copyright 2016, Wiley‐VCH.

**Figure** **2**a demonstrates the electronic structure differences between conventional semiconductors (CdS, InP) and lead halide perovskites. For conventional semiconductors, electronic surface passivation is necessary to improve PLQY such as CdSe/ZnS core/shell nanocrystals, wherein ZnS shell passivates CdSe surface to obtain approximately unity PLQY, while lead halide perovskites do not require any surface passivation to get bright photoluminescence.^[^ ^26^ ^]^ The former is called defect intolerance, and the latter is defect tolerance.^[^ ^27^ ^]^ In other words, defect tolerance means that even if there are many structural defects in the materials, these defects will not lead to electronic traps, namely having a clean bandgap. Early in 2014, Zakutayev et al. pointed out that the valence band of semiconductors composed by antibonding states could form shallow defect levels, which did not have an effect on their properties.^[^ ^28^ ^]^ Moreover, Pandey et al. demonstrated that different orbital characteristics between valence band (VB) and conduction band (CB) might introduce the shallow trap states.^[^ ^29^ ^]^

In perovskite systems, VB has antibonding nature, whereas CB consists of spin–orbit coupling. Taking CsPbI_3_ as an example, the VB is attributed to Pb(6s)–I(5p) antibonding interaction and CB originates from Pb(6p) spin–orbit effect.^[^ ^30^ ^]^ That may explain the defect tolerance of bulk halide perovskites. For lead halide perovskite nanocrystals, the dangling bonds and surface organic ligands need to be considered, which have possibilities to form trap states. Dangling bonds are nonbonding in nature and arise between bonding and antibonding states, and bonding orbitals cannot lead to the formation of VB and CB, which suggests that a deep state in bandgap will not be formed.^[^ ^31^ ^]^ Besides, some calculated results reveal that point defects in CsPbBr_3_ only form shallow states, and the defect‐tolerance characteristic of CsPbBr_3_ nanocrystals is attributed to lacking of bonding–antibonding interaction between the CB and VB.^[^ ^19b^ ^]^ As for surface organic ligands, Infante's group combined theory models and experiments to validate that the electronic structure of perovskite nanocrystals changed slightly after removal of ligands from surface as shown in Figure 2b.^[^ ^23^ ^]^ In addition, Yan's group reported the calculated transition energy levels of point defects in MAPbI_3_ (including interstitials or antisites) had higher formation energy and gave rise to excellent defect tolerance of perovskite nanocrystals as shown in Figure 2c.^[^ ^2b^ ^]^ Under different conditions (halide‐poor or halide‐rich), the formation energy of defects varies.^[^ ^32^ ^]^ In short, these researches suggest that defect tolerance is a pivotal factor for lead halide perovskite nanocrystals with high PLQY.^[^ ^21b^ ^]^ Lead‐free halide perovskites have inferior optoelectronic performances compared with lead‐based halide perovskites, which is mainly ascribed to their very higher defect densities. Hence, exploiting lead‐free halide perovskites with defect tolerance is advantageous.^[^ ^31^, ^33^ ^]^

### 2.2 Bandgap Type

It is worthy of highlighting that bandgap type is also a crucial factor in influencing the optical characteristic of materials. Tailoring bandgap magnitude has been studied with a great deal work.^[^ ^34^ ^]^ More importantly, the type of bandgap (direct or indirect) is also a significant characteristic either for photovoltaics or light emission. It must be mentioned that the relative crystal momenta of the conduction band minimum and valence band maximum decides whether a bandgap is direct or indirect.^[^ ^35^ ^]^ As shown in Figure 2d, there is an apparent difference in direct bandgap and indirect bandgap. For direct bandgap materials, the process of absorption and recombination are only invoked by photons, which can lead to high PLQY. In comparison, there is a process involving assistant phonons in indirect bandgap materials, which can form the thermal energy to decrease PLQY during the transition process.^[^ ^36^ ^]^ In lead halide perovskites, direct bandgap characteristic is vital to obtain near‐unity PLQY.^[^ ^37^ ^]^

### 2.3 Exciton Binding Energy (*E*_b_)

Apart from defect tolerance and bandgap type, exciton binding energy is also an important factor to impact the optical characteristics of materials. An exciton refers to a pair of excited electron and hole, which exist mutual attraction via a Coulombic interaction to form a neutral quasiparticle. Exciton binding energy means that the binding ability of exciton. Excitons can be classified as Frenkel excitons and Wannier excitons according to the relationship between lattice constant (*a*) and Bohr radius (*r*_B_) as shown Figure 2e.^[^ ^25^ ^]^ Generally, Frenkel excitons have a small Bohr radius of 5 Å with a large exciton binding energy ranging from 500 to 1000 meV, whereas Wannier excitons have a large Bohr radius in concomitant with a small exciton binding energy of 10–30 meV.^[^ ^38^ ^]^ At room temperature, the exciton with a binding energy smaller than thermal energy kT (26 meV) tends to dislocate into free carriers. Free carriers are opposed to localized excitons and can freely diffuse through the host lattices to propagate the excitation energy without transporting net electric charge.^[^ ^39^ ^]^ Thus, materials with smaller exciton binding energy are more suitable for solar cells. However, based on totally different design rules, a larger exciton binding energy is required for light emission, which promotes the radiative recombination of excitons efficiently. A series of research demonstrated that exciton binding energy is higher in confined structures, such as nanocrystals (<100 nm in at least one dimension), including nanoplates, nanowires, nanorods and quantum dots. The confined structures based on dimensionally physical barriers suppress the exciton dissociation and enhance radiative recombination to improve PLQY of materials.^[^ ^40^ ^]^

Zheng et al. reported the exciton binding energy of MAPbBr_3_ nanocrystals (*E*_b_ = 0.32 eV) was 3.8 times higher than that of the corresponding bulk crystals (*E*_b_ = 0.084 eV). The higher *E*_b_ contributed to enhanced PLQY (<0.1% for bulk crystals).^[^ ^41^ ^]^ Simultaneously, structurally formed potential barriers embedded in the crystal lattice, namely, low‐dimension perovskites also have confinement effect.^[^ ^42^ ^]^ Besides, it is worth noting that low‐dimension perovskites (see below) at the molecular level possess larger exciton binding energies, whose amplification is attributed to not only the quantum confinement effect but also the dielectric enhancement.^[^ ^43^ ^]^ The organic layers with low dielectric constant have poor screening effect on the attraction between electrons and holes in the inorganic layers, which can improve the exciton binding energy as experimentally validated.^[^ ^44^ ^]^

Early research reported that exciton binding energy can be improved up to four times when exciton is confined in two dimensions.^[^ ^45^ ^]^ Quantum well structure in 2D perovskites confines electrons and holes within the well, leading an improvement of larger exciton binding energies and radiative recombination due to the very different dielectric constants of the “well” and “barrier” as shown in Figure 2f.^[^ ^11^ ^]^ Ishihara showed a huge change of exciton binding energy which ranged from 1.633 eV for 3D perovskites to 3.42 eV for 0D perovskites and demonstrated the effect of smaller dielectric constant of barrier layers.^[^ ^46^ ^]^ Additionally, some research manifested that the halide and the thickness of the inorganic layers had some influences on exciton binding energy and bandgap.^[^ ^47^ ^]^ One research reported *E*_b_ = 220 meV and *E*_g_ = 2.58 eV for PEA_2_PbI_4_ in comparison with *E*_b_ = 356 meV and *E*_g_ = 3.40 eV for PEA_2_PbBr_4_ (PEA = C_6_H_5_CH_2_CH_2_NH_3_^+^).^[^ ^48^ ^]^ Nurmikko's group reported that PEA_2_(MA)*_n_*_‐1_Pb*_n_*I_3_ *_n_*_+1_ family had different bandgap and exciton binding energy with different thickness of inorganic layers.^[^ ^48^ ^]^ In summary, a larger exciton binding energy based on confined structures is considered to be beneficial to light emission. However, every coin has two sides. Auger recombination increases with the increase of exciton binding energy. Compared with 3D perovskites, the rate of Auger recombination is improved in 2D perovskites.^[^ ^49^ ^]^ In order to illustrate the corresponding effects, **Table** **2** summarizes the characteristics of defect tolerance, bandgap type and exciton binding energy of some halide perovskites.

**Table 2:** Defect tolerance, bandgap type, and exciton binding energy (*E*_b_) of some halide perovskites (NCs, nanocrystals; SCs, single crystals; BCs, bulk crystals)

Material	Defect tolerance	Bandgap type	*E*_b_ [meV]	PLQY [%]
CsPbCl_3_ NCs	Yes	Direct	67,^[^ ^50^ ^]^ 75^[^ ^4b^ ^]^	96.5,^[^ ^51^ ^]^ 97,^[^ ^6b^ ^]^ 98^[^ ^6c^ ^]^
CsPbBr_3_ NCs	Yes	Direct	47,^[^ ^50^ ^]^ 40^[^ ^52^ ^]^	96,^[^ ^6a^ ^]^ 97,^[^ ^6b^ ^]^ 100^[^ ^53^ ^]^
CsPbI_3_ NCs	Yes	Direct	25,^[^ ^50^ ^]^ 20^[^ ^4b^ ^]^	95,^[^ ^54^ ^]^ 96,^[^ ^6b^ ^]^ 100^[^ ^55^ ^]^
(OCTAm)_2_SnX_4_	–	Direct	–	95 ± 5^[^ ^56^ ^]^
Cs_3_Bi_2_Br_9 _NCs	Yes^[^ ^57^ ^]^	–	210.7	19.4^[^ ^58^ ^]^
MA_3_Bi_2_Br_9_‐Cl NCs	Yes^[^ ^57^ ^]^	Direct	259.1	54.1^[^ ^59^ ^]^
Cs_2_AgIn_0.9_Bi_0.1_Cl_6_ NCs	–	Direct	496	36.6^[^ ^24^ ^]^
Cs_2_AgInCl_6_ BCs	–	Direct	250	<0.1^[^ ^60^ ^]^
Cs_2_Ag_0.6_Na_0.4_InCl_6_:0.04Bi^3+^ BCs	–	–	–	86 ± 5^[^ ^60^ ^]^
Cs_3_Cu_2_I_5 _SCs	–	Direct	490	91.2^[^ ^61^ ^]^
Cs_2_AgBiCl_6_ NCs	–	Indirect	–	6.7^[^ ^62^ ^]^
Cs_2_AgBiBr_6_ NCs	–	Indirect	–	0.7^[^ ^62^ ^]^
Cs_2_AgBiI_6_ NCs	–	Indirect	–	<0.1^[^ ^62^ ^]^

In addition to the above discussion, there is one exceptional case that deserves more attention. Cs_2_AgInCl_6_ with direct bandgap displays an extremely low PLQY, whereas the PLQY of Cs_2_Ag_0.6_Na_0.4_InCl_6_:0.04Bi^3+^ reaches 86% after introducing Na^+^ and Bi^3+^ ions. The underlying mechanism bringing difference is worth considering developing excellent lead‐free halide perovskite materials with superior performance. There are two main reasons for the low PLQY of halide double perovskites Cs_2_AgInCl_6_. On the one hand, the emission of halide double perovskites Cs_2_AgInCl_6_ belongs to parity‐forbidden transitions. On the other hand, the wave function distributions of electron and hole exist small overlaps. After alloying with Na^+^, the parity‐forbidden transition was removed by breaking the inversion symmetry via substituting partial Ag^+^ ions. In addition, theoretical calculation revealed that Na^+^ alloying improved the wave function overlaps between electron and hole. After doping a small amount of Bi^3+^, the PLQY of Cs_2_Ag_0.6_Na_0.4_InCl_6_:0.04Bi^3+^ was further improved to 86% via passivation of defects.^[^ ^60^ ^]^

In conclusion, lead‐free halide perovskites with defect‐tolerance, direct‐bandgap, and larger exciton binding energy are beneficial for light emission. More importantly, some significant strategies to adjust bandgap type, passivate defects, increase exciton binding energy, break parity‐forbidden transition, and seek for defect‐tolerant lead‐free halide perovskites are in demand. Moreover, some methods have been proved to be effective and feasible, such as doping, post‐synthetic treatment, and developing low‐dimension perovskites or perovskite nanocrystals.^[^ ^5^, ^24^, ^56^, ^61^ ^]^

## 3 Design and Synthesis of Lead‐Free Halide Perovskites

### 3.1 Design of Lead‐Free Halide Perovskites

**Figure 3:**
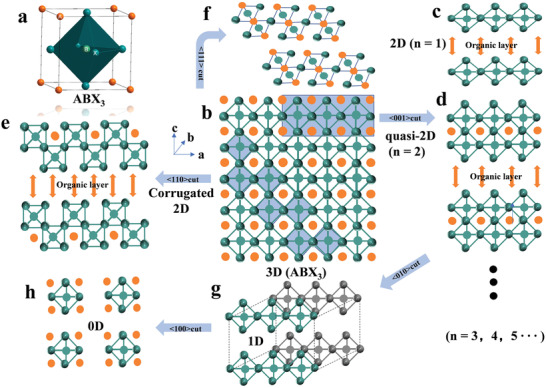
Halide perovskite structures with different dimensionalities at the molecular level. a) The unit cell of 3D perovskite. b) The <010> orientation projection of 3D halide perovskites. c) The crystal structure of <001>‐oriented 2D perovskite (*n* = 1). d) The crystal structure of <001>‐oriented quasi‐2D perovskite (*n* = 2). e) The crystal structure of <110>‐oriented 2D perovskite (*n* = 2). f) The crystal structure of <111>‐oriented 2D perovskite (*n* = 2). g) The crystal structure of 1D perovskite with octahedra connecting in a chain form. h) The crystal structure of 0D perovskites with the isolated octahedra.

Perovskite materials have a general chemical formula of ABX_3_, where A usually refers to Cs^+^, Rb^+^, CH_3_NH_2_^+^ (MA^+^) or CH(NH_2_)_2_^+^ (FA^+^), B is a divalent lead cation (Pb^2+^), and X stands for halogen (Cl^−^, Br^−^, I^−^). The common way of searching for lead‐free halide perovskites is substituting Pb^2+^ by group 14 metal elements (Sn and Ge), group 15 metal elements (Sb and Bi) and other metal elements. Notably, Goldschmidt tolerance factor (*t*) and octahedral factor (*μ*) are two important parameters to indicate the formation of perovskite structures. Here, *t* = (*r*_A_ + *r*_X_)/$\sqrt{2}$ (*r*_B_+*r*_X_) and *μ* = *r*_B_/*r*_X_, wherein *r*_A_, *r*_B,_ and *r*_X_ are the ionic radii of A, B, and X, respectively.^[^ ^63^ ^]^ Empirically, when the tolerance factor is between 0.81 and 1.11, hybrid perovskites can be formed. The octahedral factor *μ* can be used to estimate the stability of octahedra. Generally speaking, perovskite structure is stable when the octahedral factor μ ranges from 0.442 to 0.895.^[^ ^64^ ^]^ As shown in **Figure** **3**a, B cations occupy the center of octahedra, and A cations and halide anions X occupy the vertexes and face‐centers of the square in a typical 3D cubic structure, respectively.^[^ ^65^ ^]^ The ideal cubic phase is formed by arranging the corner‐sharing [BX_6_]^4−^ octahedra as shown in Figure 3b. On some occasions, perovskites may deviate from the ideal cubic phase and form a less symmetrical orthorhombic phase.^[^ ^21b^ ^]^ Lin's group have summarized five factors for tilting of BX_6_ octahedra, including Jahn–Teller effect, off‐center displacement of octahedra's central cations, small ionic radius of A, ordering of mixed cations A or B, and vacancy, and ordering of mixed anions X.^[^ ^64^ ^]^ These issues also provide us some methods to tailor the physical properties of perovskites to fulfill more applications.

To a large extent, larger A cations cannot form 3D crystal structures; they lead to warp, damage, or transform of structure to form lower‐dimensional crystals. In addition, the B cations are also considerable to keep charge neutrality. There are isovalent replacement (Sn^2+^, Ge^2+^ Cu^2+^, Y^2+^, etc.) and heterovalent replacement (Ag^+^, Cu^+^, Sb^3+^, Bi^3+^, Sn^4+^, Ge^4+^, Pd^4+^, etc.). All possible replacements of lead element are presented in Figure 1. The formulas of lead‐free halide perovskite vary as a result of the different replacements, which are AB(II)X_3_, A_2_B(IV)X_6_, A_3_B(III)_2_X_9_, A_2_B(I)B(III)X_6_, A_4_B(III)B(V)X_12_, A_4_B(II)B(III)_2_X_12_ and other perovskite derivatives.^[^ ^66^ ^]^ Double, triple, quadruple, and defect/vacancy perovskites have their general formulas of A_2_B(I)B(III)X_6_, A_3_B(III)_2_X_9_, A_4_B(III)B(V)X_12_, A_4_B(II)B(III)_2_X_12_ and A_2_B(IV)X_6_, respectively. These different types diversify the halide perovskites. Notably, the effects of mixed charges in the B sites are multiple. First, it can change the bandgap magnitudes and bandgap types. Second, it can change the optical characteristics, such as nonemission to emission, the emission color, and the emission range. Third, it can also improve the stability of perovskites.

Halide perovskites can be categorized as 3D, 2D, 1D, and 0D at the molecular level according to different arrangements of metal halide octahedra [BX_6_]^4−^ as shown in Figure 3. When smaller size cations are in A site (such as Cs^+^, MA^+^ and FA^+^), metal halide octahedra [BX_6_]^4−^ can form corner‐sharing 3D networks (Figure 3b). When A is replaced by larger size organic cations, 2D structures are formed where [BX_6_]^4−^ octahedra connect in layered or corrugated sheets that are sandwiched between large organic cations. 2D and corrugated 2D organometallic halide perovskites are formed by splitting along lattice orientations <001> and <110> from 3D perovskites, which are identified as Ruddlesden–Popper perovskites. 2D Ruddlesden‐Popper halide perovskites can be expressed as (A’)_2_(A)*_n_*_‐1_B*_n_*X_3_ *_n_*_+1_,^[^ ^67^ ^]^ where A stands for small size cations, such as Cs^+^, MA^+^, and FA^+^, A’ represents large organic cations between inorganic sheets (usually with long alkyl chains or a benzene ring), B refers to bivalent metal cations (Sn^2+^ and Pb^2+^), X refers to halides, and n stands for the number of metal halide monolayer between the insulating organic layers. *n* = 1 represents single layer inorganic octahedra (strict 2D perovskites, Figure 3c), *n* = 2–5 stands for quasi‐2D perovskites (Figure 3d), and *n* = ∞ refers to 3D perovskites.^[^ ^68^ ^]^ Notably, corrugated 2D perovskites can be formed along the <110> orientation to cut the 3D perovskites, as depicted in Figure 3e.^[^ ^69^ ^]^ There are also <111>‐ oriented 2D perovskites as shown in Figure 3f. For 1D perovskites, [BX_6_]^4−^ octahedra are connected in a chain form (corner‐sharing, edge‐sharing, or face‐sharing), and the chemical formulas rely on the connecting ways of octahedra BX_6_ and the organic cations (Figure 3g).^[^ ^70^ ^]^

In 0D halide perovskites, individual metal halide octahedra are completely surrounded by organic cations and isolated from each other. The general formula is expressed as A_4_BX_6_, where A refers to monovalent organic cations and [BX_6_]^4−^ refers to isolated octahedra (Figure 3h). It is worth noting that the above‐mentioned categories are based on molecular level rather than morphological 2D nanosheets/nanoplatelets, 1D nanowires/nanorods, and 0D quantum dots. For example, morphological 0D perovskites are called as quantum dots, in which the [BX_6_]^4−^ octahedral units are connected with strong interactions via corner‐sharing to form a 3D framework.^[^ ^42b^ ^]^ Owing to the different connectivity of the [BX_6_]^4−^ octahedral units, the 0D perovskites at the molecular level display extremely different optoelectronic characteristics in comparison with the morphological 0D quantum dots. However, there are also some relationships between molecular levels and morphological levels. On the one hand, 1D and 0D organometal halide perovskites at the molecular level can be regarded as bulk assemblies of 1D quantum wires and 0D molecules/clusters, respectively. The completely isolated octahedra in these low‐dimensional 1D and 0D perovskites enable the materials to exhibit the intrinsic properties of the individual octahedron. On the other hand, the low dimensionality both at the molecular level and morphological level possess quantum confinement effect.^[^ ^42^, ^70^ ^]^ The low dimensionalities (2D, 1D, 0D) of perovskites that will be talked below refer to the molecular level.

Currently, bulk crystals, thin films, and colloidal nanocrystals of halide perovskites are all considered as potential materials for various optoelectronic applications.^[^ ^10^, ^25^, ^71^ ^]^ Based on this background, we systematically summarize their synthesis methods.

### 3.2 Synthesis of Lead‐Free Halide Perovskites

#### 3.2.1 Synthesis of Lead‐Free Halide Perovskite Single Crystals

Single crystal can be used to analyze basic structure and physical properties of materials. It also has a low trap density and is favorable for the radiative recombination.^[^ ^72^ ^]^ Early in 1978, halide perovskite single crystals were synthesized by Weber.^[^ ^73^ ^]^ Afterwards, there was a great number of single crystal growth methods of halide perovskite reported, including temperature‐lowering crystallization (TLC), traditional solvothermal method, inverse temperature crystallization (ITC), antisolvent vapor‐assisted crystallization (AVC), and slow evaporation method (SEM).^[^ ^74^ ^]^ These methods are to obtain supersaturated precursor solution, adjust the solubility, and induce crystallization. Herein, we summarize the representative lead‐free halide perovskite single crystal growth methods.

**Figure 4:**
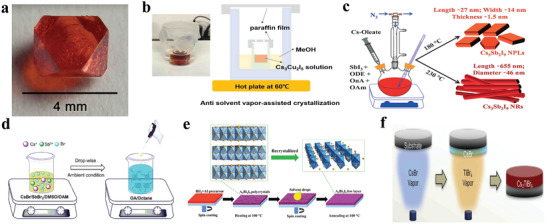
a) Photograph of a single crystal of Cs_2_AgBiBr_6_. Reproduced with permission.^[^ ^76^ ^]^ Copyright 2016, American Chemical Society. b) Schematic of antisolvent vapor assisted crystallization of Cs_3_Cu_2_I_5_ single crystal. Reproduced with permission.^[^ ^61^ ^]^ Copyright 2018, Wiley‐VCH. c) Schematic representation of the synthesis of Cs_3_Sb_2_I_9_ nanoplatelets and Cs_3_Sb_2_I_9_ nanorods. (ODE = 1‐octadecene, OnA = octanoic acid, OAm = oleylamine.) Reproduced with permission.^[^ ^96^ ^]^ Copyright 2017, Wiley‐VCH. d) Schematic illustration of the reaction system for LARP technique. Reproduced with permission.^[^ ^80^ ^]^ Copyright 2017, American Chemical Society. e) Schematic view of the synthesis process of the ultrathin A_3_Bi_2_I_9_ films (A = Cs^+^ or MA^+^). Reproduced with permission.^[^ ^95^ ^]^ Copyright 2017, Wiley‐VCH. f) Schematic illustration of the vapor‐based synthesis of Cs_2_TiBr_6_ halide perovskite thin film. Vapor‐deposited CsBr thin film before annealing (film I), after 12 h annealing (film II), and after 24 h annealing (film III) at 200 °C. Reproduced with permission.^[^ ^97^ ^]^ Copyright 2018, Cell Press.

Temperature‐lowering crystallization (TLC) is performed by accurately lowering the temperature of preferred seed solution to induce the oversaturation of the solute. Subsequently, the saturated aqueous perovskite precursor HX (X = Cl, Br, I) solution, which contains inorganic metal ions and organic halide ions, grows halide perovskite crystals slowly. 2D Sn‐based single crystals (C_4_H_9_NH_3_)_2_(CH_3_NH_3_)*_n_*_‐1_Sn*_n_*I_3_ *_n_*_+1_ (*n* = 1–5) were synthesized by temperature‐lowering crystallization method, where the cooling rate was 2–5 °C h^−1^.^[^ ^75^ ^]^ **Figure** **4**a, shows high‐quality big size Cs_2_AgBiBr_6_ single crystals.^[^ ^76^ ^]^ Su's group prepared red‐emitting 0D Cs_2_InBr_5_·H_2_O single crystals. Typically, CsBr and InBr_3_ with certain molar proportions were dissolved in HBr solution at 130 °C for 5 min. Next, the hot solution was transferred into a preheated Teflon‐lined stainless‐steel autoclave and maintained at 130 °C for 30 min. The crystals were obtained by slowly cooling the solution down to room temperature then washed and dried.^[^ ^72^ ^]^ Tao's group also presented cubic CH_3_NH_3_SnI_3_ and CH(NH_2_)_2_SnI_3_ single crystals with dimensions of 20 × 16 × 10 mm^3^ and 8 × 6 × 5 mm^3^ by the same method. Taking CH_3_NH_3_SnI_3_ for example, a certain amount of SnO and CH_3_NH_3_I were dissolved in a mixture of HI and H_3_PO_2_ to form a clear solution. Notably, H_3_PO_2_ acted as a reducing agent to stabilize Sn^2+^ and I^−^ ions.^[^ ^77^ ^]^ 0D (C_8_NH_12_)_4_Bi_0.57_Sb_0.43_Br_7_∙H_2_O single crystals were also grown similarly.^[^ ^78^ ^]^ Rb_7_Bi_3_Cl_16_ single crystals were synthesized by a traditional hydrothermal method with a temperature‐lowering process. The freezing rate of 2 °C h^−1^ was crucial to the growth of these single crystals.^[^ ^79^ ^]^ The temperature‐lowering method is a simple and stable process to grow high‐quality big size single crystals. However, this method is not appropriate for materials with low solubility at high temperatures. In this condition, inverse temperature crystallization (ITC) method is suitable. Song's group reported that Cs_3_Sb_2_Br_9_ single crystals were grown by ITC method, where CsBr and SbBr_3_ with certain proportions were dissolved in DMSO at room temperature. Then, the solution was filtered with 0.2 mm pore size. In the end, the filtrates were placed in a vial and was kept in a 90 °C oil bath for 3 h to obtain the final product.^[^ ^80^ ^]^ It should be noted that ITC method may generate more defects, because the grow rate of single crystal is difficult to control.

Antisolvent vapor‐assisted crystallization (AVC) is appropriate for materials with high solubility in some solvents but poor solubility in other solvents. Herein, an appropriate antisolvent is chosen to diffuse slowly into a solution containing the precursors, which can lead to the crystallization of halide perovskites. Abulikemu et al. grew (CH_3_NH_3_)_3_Bi_2_I_9_ single crystals by AVC. First, CH_3_NH_3_I and BiI_3_ with certain ratios were dissolved in *γ*‐butyrolactone, and the above reaction vessel was placed in an antisolvent (anhydrous dichloromethane) without any direct contact. Then, antisolvent diffused to the CH_3_NH_3_BiI_3_ solution after one day, which changed the solubility and thus promoted the growth of single crystals.^[^ ^81^ ^]^ Cs_3_Cu_2_I_5_ single crystals were also synthesized similarly. As shown in Figure 4b, filtered Cs_3_Cu_2_I_5_ solution was injected in a vial and wrapped with a paraffin membrane with a small hole. The vial was put in a beaker where there was methyl alcohol (MeOH) as an antisolvent. Moreover, this beaker was sealed. Notably, the paraffin membrane has two effects which not only balanced the antisolvent atmosphere but also suppressed its evaporation. Subsequently, Cs_3_Cu_2_I_5_ single crystals were obtained after the breaker was put on hotplate at 60 °C for 48 h.^[^ ^61^ ^]^ (Ph_4_P)_2_SbCl_5_ (Ph_4_P = tetraphenylphosphonium) single crystals were also synthesized similarly and demonstrated excellent optical properties.^[^ ^82^ ^]^ Generally, the crystal grow rate of AVC is relatively slow, but the quality of the obtained single crystals is decent.

Slow evaporation is also a facile and traditional method for single crystal growth. Growth of (CH_3_NH_3_)_3_Bi_2_I_9_ single crystals were reported, where the methanol solution containing CH_3_NH_3_I and BiI_3_ was ultrasonicated for ≈0.5 h, then transferred to a clean vial and evaporated slowly overnight at room temperature.^[^ ^83^ ^]^

Notably, large bulk single crystals may be more convenient when analyzing the intrinsic characteristics of materials. However, large area and thin single crystals are in demand in terms of optoelectrical device applications in favor of carrier transportation. Researchers are making great efforts to optimize the crystallization process toward simple and green processes and to control the nucleation in order to obtain proper single crystals to meet the different application requirements. Intriguingly, different facets of one single crystal demonstrate different characteristics, namely, the anisotropy phenomenon. Hence, facets engineering can be considered to exploit high‐performance single crystals.

#### 3.2.2 Synthesis of Lead‐Free Halide Perovskite Colloids

Hot injection and recrystallization are widely applied to colloidal nanocrystal synthesis either for lead‐based or lead‐free halide perovskites.^[^ ^33^, ^84^ ^]^ Figure 4c shows the synthesis of Cs_3_Sb_2_I_9_ via hot injection. SbI_3_ was dissolved in a mixture of surfactants and solvent mostly with oleic acid (OA), oleylamine (OLA) and 1‐octadecene (ODE). Afterwards, the preheated Cs‐oleate precursor was injected in the aforementioned solution at a specific temperature; after reacted for a while, the synthesis was quenched using an ice bath. It is noteworthy that the size and morphology can be tailored by the injection temperature, reaction time, and precursor concentration. During the process of synthesizing Cs_3_Sb_2_I_9_ nanocrystals, a different reaction temperature leads to a different morphology. Subsequently, a lot of lead‐free halide perovskites were synthesized through hot injection method.^[^ ^84^, ^85^ ^]^ Locardi et al. utilized diphenyl ether and benzoyl chloride to prepare Cs_2_AgInCl_6_ and Mn^2+^ doped Cs_2_AgInCl_6_ double perovskite nanocrystals.^[^ ^66c^ ^]^ Zhang et al. synthesized (OAm)_2_SnBr_4_ (OAm = C_18_H_35_NH_3_) belonged to 2D structural dimensionality.^[^ ^86^ ^]^ The materials were obtained by injecting a SnBr_2_‐TOP solution into a solution containing ODE, OA and OLA at 180 °C under a N_2_ atmosphere. The reaction was maintained for 10 s and was quenched by ice water swiftly. Subsequently, the product was obtained by adding hexane and centrifuging. The XRD measurement revealed the presence of a periodic 2D structure of the (OAm)_2_SnBr_4_ perovskite with a regular interval of 2.3°.

Generally, perovskite nanocrystals synthesized via hot injection method possess uniform size and shape. However, there are also some disadvantages, including strict synthesis temperature, not suitable for mass production, and inert gas atmosphere protection. Apart from hot injection method, reprecipitation (RP), including ligand‐assisted reprecipitation (LARP) and antisolvent reprecipitation (ASRP), is also of great interest due to their facile and high‐yield fabrication. In 2016, Zeng's group reported that CsPbX_3_ (X = Cl, Br, I, or a mixture of them) perovskite nanocrystals were synthesized via LARP and elaborated the relevant dissolution, supersaturation and rapid recrystallization mechanism.^[^ ^52^ ^]^ There was also a great number of lead‐free halide perovskite materials synthesized by RP method. Typically, Cs_3_Sb_2_Br_9_ nanocrystals were synthesized by modified LARP. Zhang et al. first examined the solubility of CsBr and SbBr_3_ in typical solvents. Then they found that the most suitable solvents were *N,N*‐dimethylformamide (DMF) or dimethyl sulfoxide (DMSO), and the antisolvent was octane (Figure 4d). During the synthesis process, OA and OLA served as surface ligands to control the morphology and crystallization.^[^ ^80^ ^]^ In most RP synthesis process, DMSO and DMF often serve as universal solvents while the choices of antisolvent are diverse, such as toluene, isopropanol, ethanol, and octane.^[^ ^24^, ^59^ ^]^ Han's group reported a series of lead‐free halide double perovskites synthesized via RP, where isopropanol was chosen as an antisolvent. Typically, to synthesize Cs_2_AgIn_0.9_Bi_0.1_Cl_6_, CsCl, AgCl, InCl_3_, and BiCl_3_ with a certain proportion were dissolved in DMSO to form a precursor solution. Then this precursor solution was injected into isopropanol under vigorous stirring. Finally, the product was obtained by centrifugation. If some OA was previously added in isopropanol, then OA‐capped halide double perovskite nanocrystals were obtained. Experiments showed that OA‐capped halide double perovskite nanocrystals displayed superior emission properties than OA‐free nanocrystals. That was attributed to the passivation effect of OA and that promoted the radiative recombination.^[^ ^24^ ^]^

There are other ways to synthesize lead halide perovskite colloids. However, for the lead‐free halide perovskite colloid system, only the above two methods have been reported. We believe other methods and new techniques will be used for the synthesis of lead‐free halide perovskite colloids.

#### 3.2.3 Synthesis of Lead‐Free Halide Perovskite Thin Films

The qualities of thin films are vital to obtaining optoelectronic devices with excellent performance. The quality of thin films is influenced by many synthetic factors, including the choice of solvent, precursor, reagent's concentration and ratio, annealing and drying conditions, and deposition sequence. The smooth, uniform, and hole‐free thin films are in demand. Several reviews summarized the synthetic approaches of lead halide perovskite thin films.^[^ ^13^, ^87^ ^]^ Herein, we summarize some representative preparation methods for lead‐free halide perovskite thin films.

Spin‐coating and vapor deposition are two common approaches. To obtain the films fabricated by spin‐coating method, metal halide salts (such as SnBr_2_, BiBr_3_) and organic or inorganic halide salts (such as MABr, CsBr, CsI) were dissolved in an organic solvent (DMF, DMSO, GBL (*γ*‐butyrolactone), NMP (n‐methylpyrrolidone), ACN (acetonitrile) or a mixture of them) to form a precursor solution, and then it was spin‐coated on substrates along with thermal annealing process. It is worth exploring the processing time and temperature for different precursor composition to get high‐quality thin films.

Cs_2_AgBiBr_6_ thin films were reported for the first time by Bein’ s group in 2017. CsBr, AgBr, and BiBr_3_ were dissolved in DMSO to form a precursor solution at 75 °C, and the substrate was also kept at 75 °C. Then precursor solution was spin‐coated on the substrate at 2000 rpm for 30 s, subsequently annealed at 285 °C for 5 min to get double perovskite phase thin films.^[^ ^88^ ^]^ To get higher‐quality Cs_2_AgBiBr_6_ thin films, Ning et al. reported that Cs_2_AgBiBr_6_ single crystals were dissolved in DMSO under a temperature range from 100 to 130 °C. After a complete dissolution, the solution was spin‐coated onto the substrate at 3000 rpm for 40 s at room temperature. The obtained thin films were annealed at 250 °C for 5 min to crystallize further.^[^ ^89^ ^]^ The thickness of the film was adjusted by varying the concentration of the precursors.^[^ ^90^ ^]^ The annealing temperature is also vital for the thin film's quality. Sun's group fabricated CsSnI_3_ thin films under different annealing temperatures. Increasing the annealing temperature made a coarse grain size.^[^ ^91^ ^]^

Antisolvent washing is also considered to be an important procedure to fabricate perovskite thin films in the solution process.^[^ ^92^ ^]^ Yu et al. reported that Cs_3_Bi_2_I_9‐_ *_x_*Br*_x_* perovskite thin films were fabricated by further antisolvent washing. Chlorobenzene was utilized as the antisolvent in this process. The thin films were fabricated via depositing the precursor, then chlorobenzene was drop‐cast on them and the films were annealed at 200 °C for 10 min. There was a huge difference with and without chlorobenzene treatment. The surface morphologies consist of grains, and the thin films became more compact and dense after chlorobenzene treatment.^[^ ^93^ ^]^ In the antisolvent method, the solubility of the precursors in solvent and antisolvent has a substantial influence on the crystallization process composed of nucleation and growth process. Therefore, the choice of solvent and anti‐solvent is pivotal.^[^ ^94^ ^]^

As shown in Figure 4e, a simple spin‐coating method was applied to prepare the ultrathin nanosheets of A_3_Bi_2_I_9_. Briefly, a precursor solution containing CsI (or MAI) and BiI_3_ was deposited on a glass substrate via spin‐coating and heating to obtain A_3_Bi_2_I_9_ thin films. In order to improve the quality, a dissolution‐recrystallization process was applied, which make the thin films good crystallinity compared with the simple spin‐coating only. A small amount of polar organic solvents, CH_3_OH and DMF were then spin‐coated onto the A_3_Bi_2_I_9_ thin films to assist recrystallization. Finally, the sample was annealed to remove the solvent, and the ultrathin films were thus formed.^[^ ^95^ ^]^

Two‐step deposition also plays an important role in improving thin film's morphology. Panthani's group reported that A_3_Bi_2_I_9_ (A= FA^+^, MA^+^, Cs^+^ or Rb^+^) thin films were fabricated with it. First, a certain amount of BiI_3_ dissolved in a mixture of tetrahydrofuran (THF) and DMSO was deposited onto the substrate, and spin coated at a certain temperature for some time in concomitant with annealing. Then AI solution was spin coated onto the BiI_3_ thin films and followed by annealing. In comparison with one‐step deposition, A_3_Bi_2_I_9_ thin films synthesized by two‐step deposition are smoother and more homogeneous in grain size.^[^ ^98^ ^]^

Vapor‐based deposition technique is another common approach in fabricating halide perovskite thin films.^[^ ^99^ ^]^ As shown in Figure 4f, Cs_2_TiBr_6_ thin films were synthesized via vapor deposition.^[^ ^97^ ^]^ The CsBr thin films predeposited onto the substrate were annealed in a TiBr_4_ vapor atmosphere at 200 °C. In this process, annealing temperature and annealing time were deterministic. Chen et al. fabricated highly stable and efficient CsSn_0.5_Ge_0.5_I_3_ thin films by single‐source evaporation method.^[^ ^100^ ^]^ Wu's group fabricated CsSnBr_3_ thin films via vacuum vapor deposition, which endowed the thin films with a grain size of 60 nm.^[^ ^101^ ^]^ Wang et al. presented a sequential vapor deposition procedure to fabricate double perovskite Cs_2_AgBiBr_6_ thin films with large grain sizes and uniform and smooth surface.^[^ ^102^ ^]^

Spin‐coating and vapor deposition techniques have their own advantages and disadvantages. Spin‐coating method is a low‐cost synthesis but not suitable for mass production. Vapor‐based deposition method presents apparent advantages for large area preparation and controllability.

In addition to the spin‐coating and vapor deposition techniques, some particular fabrication methods were also developed to fabricate thin films. Gao's team utilized a different ultralow vacuum deposition route to fabricate highly compact, pinhole‐free and large‐grained MA_3_Bi_2_I_9_ thin films, which is a gas‐solid reaction circumventing any solvent contact.^[^ ^103^ ^]^ Yokoyama et al. also developed a controlled gas‐solid reaction to fabricate MASnI_3_ thin films with excellent uniformity.^[^ ^104^ ^]^ Yang's group reported Sn‐based high‐quality perovskite thin films with large‐area via a green solution process. A gas pump is applied during the fabricating thin films by lowering the pressure so to remove the solvent quickly.^[^ ^105^ ^]^ In addition, PHABiI_4_ ( PHA = C_6_H_5_NH_3_^+^) thin films were synthesized by the above method when the pressure ranged from 1500 to 30 000 Pa, which displayed superior humidity stability up to 330 d.^[^ ^105a^ ^]^

It is worth highlighting that perovskite single‐crystal thin films raise wide attention in various optoelectronic applications.^[^ ^106^ ^]^ They possess the characteristics of both single crystals and polycrystalline thin films. Compared with polycrystalline thin‐films and nanocrystals, perovskite single crystals have lower defect densities, higher carrier mobility and longer diffusion lengths owing to the absence of grain boundary. However, inefficient carrier transport and accumulation behavior will take place due to the difference between short carrier diffusion length and perovskite single crystal thickness. In order to solve the above problems, perovskite single‐crystal thin films with proper thickness (e.g., < 200 *μ*m) are getting more attention. So far, lead‐free halide perovskite single‐crystal thin films have not been reported.

## 4 Structural and Optical Properties of Lead‐Free Halide Perovskite Materials

**Figure 5:**
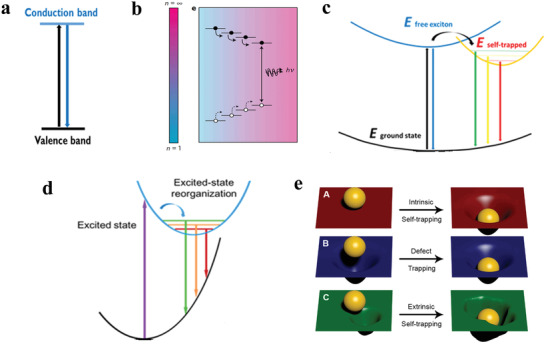
a) Direct band‐edge emission mechanism for 3D perovskites. b) Quasi‐2D multiphase perovskite materials with different 〈*n*〉 values channel energy which concentrate to smallest bandgap emitters. Reproduced with permission.^[^ ^107^ ^]^ Copyright 2016, Springer Nature. c) Mechanism of free exciton and self‐trapped exciton emission in corrugated 2D, and 1D perovskites. d) Mechanism for emission from a reorganized excited state in 0D perovskites. a,c,d) Reproduced with permission.^[^ ^70^ ^]^ Copyright 2017, American Chemical Society. e) Exciton intrinsic self‐trapping and exciton extrinsic self‐trapping presented by a sheet and hard ball model. Reproduced with permission.^[^ ^109b^ ^]^ Copyright 2018, American Chemical Society.

Some reported researches manifest that the photophysical properties are exceedingly various for halide perovskites with different dimensionalities. We here summarize common photophysical processes and characteristics of halide perovskites with different dimensionalities at the molecular level. 3D, quasi‐2D, and 2D halide perovskites for light generation generally demonstrate small Stokes shifts, narrow FWHM, and lifetime on the order of nanoseconds. These stem from the direct excited‐state transitions, namely the recombination of free excitons. The process is that electrons transit from the ground state to the excited state, generating holes in the ground state under the light excitation. Then electrons and holes recombine radiatively for light emission, as shown in **Figure** **5**a.^[^ ^70^ ^]^ Especially for quasi‐2D halide perovskites, there is more than one phase. The multiphase causes channel energy transfer across an inhomogeneous energy landscape, concentrating carriers on smaller bandgap emission (Figure 5b).^[^ ^107^ ^]^

Unlike typical narrow emission of 3D and 2D, corrugated 2D and 1D perovskites show broad‐band emissions with large stokes shifts. The broad emission is attributed to the self‐trapped excitons (STEs), and the narrow band is attributed to the free excitons (Figure 5c).^[^ ^70^, ^108^ ^]^ Besides, 0D perovskites show totally self‐trapped excitons emission (Figure 5d).^[^ ^70^ ^]^ Self‐trapped excitons refer to an electron–hole pair bound in the defects, which are from transient lattice deformation. It is noteworthy that STEs are viewed as “excited state defects” because there exists strong exciton‐lattice coupling in the excited state.^[^ ^66b^ ^]^ Transient absorption spectrum can provide direct identification for self‐trapped excitons.^[^ ^109b^ ^]^ Self‐trapped excitons can be categorized as intrinsic self‐trapping and extrinsic self‐trapping according to the ways the defects formed. A model using a hard ball as electron or hole and a soft sheet as deformable lattice can depict intrinsic self‐trapping and extrinsic self‐trapping (Figure 5e). Figure 5e‐A shows that the perfect sheet is distorted due to the existence of the hard ball, whereas the sheet can return to its original state in the absence of the hard ball. Figure 5e‐B exhibits a different condition that initially distorted sheet exists. Figure 5e‐C illustrates that an extrinsic self‐trapping is relevant to the local nonuniformity of the lattice.^[^ ^109b^ ^]^ Notably, a great deal of researches suggest that the presence of self‐trapped state is relevant to the dimension of materials, and low dimensional materials are beneficial to the formation of self‐trapped excitons.^[^ ^110^ ^]^ Generally, at low temperatures, weak self‐trapped exciton emissions can be seen for 3D halide perovskites. At room temperature, free exciton and self‐trapped exciton emissions for 2D and 1D perovskites can be observed. While 0D perovskite structure is a suitable environment to form self‐trapped states as a result of the absence of the potential energy barrier between the self‐trapped excited states and the free exciton.^[^ ^70^ ^]^ Remarkably, the Huang‐Rhys factor can be used to judge the soft crystal nature of materials, and the softer crystal nature could more easily generate the self‐trapped exciton emissions.^[^ ^111^ ^]^

There are several fundamental requirements for other metals to substitute lead in halide perovskites, including the coordination type, ionic valence, and ionic radius.^[^ ^112^ ^]^ Generally, the sixfold coordination is required to form the perovskite structure. Typically, Sn^2+^, Ge^2+^, Bi^3+^, Sb^3+^ all possess sixfold coordination. In addition, the lead‐free halide perovskites also must be electrically neutral. In ABX_3_ perovskite structure, B should be bivalent. So some typical bivalent ions, such as group 14 metals of Sn^2+^ and Ge^2+^ are the excellent candidates.^[^ ^33^, ^113^ ^]^ Notably, two bivalent B^2+^ can be substituted by B^+^ and B^3+^ to form halide double perovskites, or vacancy and B^4+^ to form vacancy‐ordered halide double perovskites.^[^ ^66^ ^]^ In some perovskite derivatives, B can also be trivalent, like group 15 metals of Bi^3+^ and Sb^3+^.^[^ ^58,^, ^66b^ ^]^ Notably, some possible substitutes of lead element have been demonstrated in Figure 1. Especially, the ionic radius is also a significant factor, determining the stability and symmetry of the perovskite structure. The ionic radius of Pb^2+^ is 1.19 Å, and lead in perovskites can be substituted by several metal elements with similar ionic radius, including Sn^2+^(1.02 Å), Ge^2+^(0.73 Å), Bi^3+^(1.03 Å), Sb^3+^(0.76 Å), and Sn^4+^(0.69 Å), according to the Goldschmidt's tolerance factor (*t*).^[^ ^114^ ^]^ In the following sections, the optical and optoelectronic properties of lead replaced by the above‐mentioned metals will be deeply discussed.

### 4.1 Sn‐Based Halide Perovskites

Tin (Sn) and Pb are in the same group of the periodic table, and Sn has some similar properties to Pb, including similar ion radii (119 pm for Pb^2+^ and 112 pm for Sn^2+^). Hence, Sn is a preferential element for lead‐free halide perovskites. Remarkably, Sn is less much toxic than Pb. Unfortunately, Sn^2+^ is prone to oxidation forming its tetravalent state, which leads to a high defect density and generates trap states, thereby lowering the radiative recombination. The P‐type self‐doping can be introduced in decomposed materials as a result of the presence of Sn^4+^. The phenomenon of self‐doping will lead to perovskite materials with metal‐like properties, which is unbeneficial for device performance. More specifically, Mitzi et al. manifested that low‐dimensional Sn‐based halide perovskites could efficiently suppress the metallic conductivity of 3D organic tin halide perovskites.^[^ ^75^ ^]^ Sn‐based halide perovskites with different dimensions at the molecular level have been applied widely to photovoltaics, field‐effect transistors, LEDs, lasers and photocatalysis.^[^ ^115^ ^]^ Herein, we summarize the structural and optical properties of Sn‐based halide perovskites with different dimensionalities.

#### 4.1.1 3D Sn‐Based Halide Perovskites

**Figure 6:**
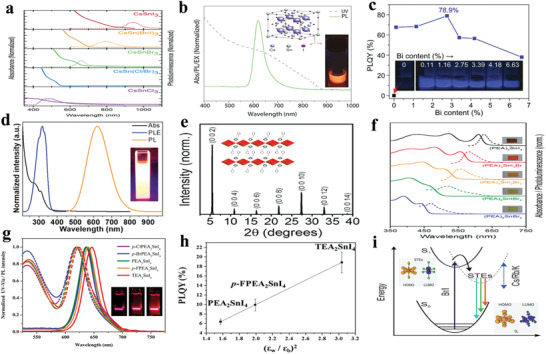
a) Absorption and steady‐state PL spectra of CsSnX_3_ (X = Cl, Cl_0.5_Br_0.5_, Br, Br_0.5_I_0.5_, I) nanocrystals. Reproduced with permission.^[^ ^33b^ ^]^ Copyright 2016, American Chemical Society. b) Absorption and PL spectra of Cs_2_SnI_6_ nanobelts under ambient conditions. Inset: a photograph of the colloidal solution of the Cs_2_SnI_6_ nanobelts under the excitation of 365 nm and the crystal structure of Cs_2_SnI_6_. Reproduced with permission.^[^ ^70^ ^]^ Copyright 2016, American Chemical Society. c) PLQY of Cs_2_SnCl_6_:*x*Bi^3+^ with *x* of 0%, 0.11%, 1.16%, 2.75%, 3.39%, 4.18%, and 6.63%. Inset: images of Cs_2_SnCl_6_:*x*Bi^3+^ under 365 nm UV light illumination at room temperature. Reproduced with permission.^[^ ^16e^ ^]^ Copyright 2018, Wiley‐VCH. d) Normalized absorption, PLE (photoluminescence excitation) monitored at 620 nm, and PL spectra of the (OAm)_2_SnBr_4_ perovskite thin films. Inset: photograph of the colloidal suspension of (OAm)_2_SnBr_4_ perovskites under the excitation of UV light. Reproduced with permission.^[^ ^86^ ^]^ Copyright 2019, American Chemical Society. e) Powder X‐ray diffraction patterns of (PEA)_2_SnI_4_ perovskite thin films. Inset: schematic of (PEA)_2_SnI*_x_*Br_4−_ *_x_* perovskite crystal structure. f) Normalized absorption and PL spectra of (PEA)_2_SnI_4_, (PEA)_2_SnI_3_Br, (PEA)_2_SnI_2_Br_2_, (PEA)_2_SnIBr_3,_ and (PEA)_2_SnBr_4_ perovskite thin films processed on glass from top to bottom. Inset: image of corresponding samples for different compositions. Reproduced with permission.^[^ ^129^ ^]^ Copyright 2017, American Chemical Society. g) Absorption and PL spectra (*λ*_ex_ = 375 nm) of tin perovskite nanodisks in toluene. Inset: photos of PEA_2_SnI_4_ (left), p‐FPEA_2_SnI_4_ (center), and TEA_2_SnI_4_ (right) nanodisks prepared under illumination with a 375 nm. h) Correlation plot of PLQY versus (*ε*_w_/*ε*_b_)^2^. *ε*_w_ and *ε*_b_ refer to the dielectric constants of well layers and barrier layers. Reproduced with permission.^[^ ^44^ ^]^ Copyright 2019, American Chemical Society. i) Configurational coordinate diagram of the self‐trapping excitons in Cs_4−_ *_x_*A*_x_*Sn(Br_1−_ *_y_*I*_y_*)_6_ (A = K, Rb). Inset: ground‐state and excited‐state (STE) HOMOs and LUMOs. Reproduced with permission.^[^ ^133^ ^]^ Copyright 2018, Wiley‐VCH.

3D Sn‐based halide perovskites were first synthesized in 1974 for CsSnX_3_ single crystals.^[^ ^116^ ^]^ Later on, Sn‐based halide perovskite materials in the form of thin films and other bulk materials were intensively synthesized and investigated, especially for solar cell applications. Considerable research efforts have been devoted to the PL behaviors of 3D Sn‐based halide perovskites. At room temperature, solid‐state MASnI_3_ with a direct bandgap shows a strong PL emission at 950 nm, which corresponds to the onset of the absorption edge.^[^ ^16b^ ^]^ MA was substituted by ethylenediammonium (en) and formamidinium (FA) to form new materials. Their thin films displayed different emission wavelengths at about 870, 840, and 760 nm with 0, 10, and 25% en/FA ratio. The introduction of en opened up a new bandgap tuning mechanism that originated from massive Schottky style defects.^[^ ^117^ ^]^ As shown in **Figure** **6**a, the absorbance and steady‐state PL of all‐inorganic CsSnX_3_ (X = Cl, Cl_0.5_Br_0.5_, Br, Br_0.5_I_0.5_, or I) perovskite nanocrystals were demonstrated. The spectra can be adjusted from visible to near‐infrared by changing the halogens. The PL mechanism is assigned to a fast band‐edge emission and a slow radiative recombination at shallow intrinsic defect sites.^[^ ^33b^ ^]^ An early research also confirmed that the formation energy of defects was so low (250 meV) that presented high defect densities.^[^ ^118^ ^]^ Chen et al. showed that CsSnX_3_ quantum rods synthesized via a simple solvothermal method showed composition‐tunable PL from 625 to 709 nm with a FWHM of 32 nm under the excitation of 532 nm.^[^ ^119^ ^]^ Interestingly, CsSnBr_3_ hollow nanocages synthesized by the hot injection method presented an absorption onset at 655 nm and PL emission at 685 nm.^[^ ^120^ ^]^ Wu et al. demonstrated that the substitution of B site in CsSnCl_3_ nanocrystals and thin films with In^3+^ or Mn^2+^ ions displayed cyan (484 nm) and red emission colors (645 nm), respectively. The PL mechanisms were ascribed to B‐site vacancies and the energy transfer between CsSn_0.9_Cl_3_ and Mn^2+^, respectively.^[^ ^121^ ^]^ It was also demonstrated that the oxidation of Sn^2+^ to Sn^4+^ was suppressed to a large extent in virtue of reducing agents.^[^ ^122^ ^]^ To address the stability of Sn^2+^ essentially, Sn^4+^‐based Cs_2_SnI_6_ nanocrystals with different morphologies were synthesized via a simple phosphine‐free hot‐injection method.^[^ ^123^ ^]^ The emission peak at 620 nm did not shift under different excitation wavelengths, as shown in Figure 6b, which suggested that the emission was originated from Cs_2_SnI_6_ nanocrystals.^[^ ^70^ ^]^ Han et al. reported that Cs_2_SnX_6_ (X = Br and I) applied to photodetectors showed two PL peaks at 673 and 870 nm.^[^ ^115b^ ^]^ It can be seen from Figure 6c that Cs_2_SnCl_6_: Bi^3+^ materials are blue phosphors with a photoluminescence quantum yield (PLQY) of 78.9%, which is comparable with blue‐emitting lead halide perovskites. Cs_2_SnCl_6_ is a nonluminous material, and the bandgap decreases with Bi^3+^ doping. Density functional theory (DFT) suggests that the thermodynamically preferred [Bi_Sn_+V_Cl_] defects are accounted for the optical absorption and blue emission. And the electronic band structure confirmed the valence band of Bi^3+^ doped Cs_2_SnCl_6_ consisted of Bi 6s and Cl 2p orbitals.^[^ ^16e^ ^]^

#### 4.1.2 2D Sn‐Based Halide Perovskites

In 1993, Papavassiliou et al. synthesized PEA_2_SnI_4_ and PEA_2_SnBr_4_ 2D halide perovskites.^[^ ^124^ ^]^ Subsequently, Mitzi et al. displayed <100>‐oriented (C_4_H_9_NH_3_)_2_(CH_3_NH_3_)*_n_*_‐1_Sn*_n_*l_3_ *_n_*_+1_ layered halide perovskites and single crystals of <110>‐oriented [NH_2_C(I=NH_2_]_2_(CH_3_NH_3_)*_m_*Sn*_m_*I_3_ *_m_*_+2_ layered perovskites.^[^ ^75^, ^125^ ^]^ These researches demonstrated that the choices of different organic cations could lead to different crystallographic orientations of the perovskite sheets.^[^ ^75^, ^125^ ^]^ In recent years, 2D halide perovskites have become a striking research spotlight. It is noteworthy that low‐dimensional Sn‐based halide perovskites exhibit remarkably enhanced air stability in comparison with their 3D counterparts.^[^ ^126^ ^]^ (OAm)_2_SnBr_4_ 2D layered perovskites showed bright orange emission centered at 620 nm with PLQY of 88% and FWHM of 140 nm (Figure 6d).^[^ ^86^ ^]^ It is worth noting that 2D layered (OCTAm)_2_SnX_4_ (OCTAm = octylammonium cation) synthesized in aqueous phase has a high absolute PLQY of near‐unity in the solid‐state with PL emission centered at 600 nm and a broad bandwidth of 136 nm.^[^ ^56^ ^]^ These broad emission is attributed to the self‐trapped state emission of tin‐layered perovskites.^[^ ^108^ ^]^ However, for display applications, narrow band emissions are preferred to obtain high color purity. Weidman et al. reported *n* = 1 and *n* = 2 (*n* refers to the layers of metal halide octahedra) Sn‐based 2D perovskite nanoplates with narrow band emissions.^[^ ^127^ ^]^ Strongly coupled 2D Sn‐based halide perovskite nanoplates PEA_2_SnI_4_ demonstrated an emission at 640 nm, with a PLQY of 6.40 ± 0.14% and FWHM as small as 36 nm. Moreover, the investigation also suggested that aliphatic carboxylic acid was found to play a crucial role in reducing the tin perovskite defect density, thereby improving the emission intensity and stability of tin halide perovskite nanoplates.^[^ ^128^ ^]^

XRD with a periodic diffraction pattern in Figure 6e confirmed the 2D structure of (PEA)_2_SnI*_x_*Br_4‐_ *_x_* thin films. (PEA)_2_SnI*_x_*Br_4‐_ *_x_* were deposited under inert atmosphere and showed superior PL properties with the tunable wavelengths from 650 to 450 nm by adjusting the I/Br ratio (Figure 6f).^[^ ^129^ ^]^ Chiu's group reported a series of 2D layered Sn‐based perovskites to unveil the effects of dielectric confinement on PL. Figure 6g shows the absorption and PL spectra of 2D Sn‐based halide with different A sites. Especially, as shown in Figure 6h, the PLQY of 2D Sn‐based perovskites with different A sites varied linearly with the ${\left(\frac{\varepsilon_{w}}{\varepsilon_{b}}\right)}^{2}$, which can ascribe to the impact of the exciton binding energy. In the formula $E_{b}^{2D}=4{\left(\frac{\varepsilon_{w}}{\varepsilon_{b}}\right)}^{2}E_{b}^{3D}$, the *ε*_w_ and *ε*_b_ are the dielectric constants of inorganic and organic layers, respectively; $E_{b}^{2D}$ and $E_{b}^{3D}$ are the exciton binding energies of 2D halide perovskite and corresponding 3D halide perovskite, respectively.^[^ ^48b^ ^]^ According to this formula, we know that the dielectric constants of inorganic layers and organic layers have an influence on the exciton binding energy of 2D halide perovskites. The dielectric confinement using different aromatic organic cations was confirmed in the investigation. Furthermore, the research demonstrated that nanoscale TEA_2_SnI_4_ (TEA = thienylethylamine ) layered perovskites showed a record high PLQY of 21% with FWHM of 32 nm.^[^ ^44^ ^]^

#### 4.1.3 1D and 0D Sn Based Perovskites

In addition to the 2D Sn‐based halide perovskites, 1D and 0D Sn‐based halide perovskites have also been studied recently.^[^ ^130^ ^]^ Crystals of 0D (C_4_N_2_H_14_X)_4_SnX_6_ (X = Br, I) and 1D (C_4_N_2_H_14_)SnBr_4_ were synthesized. The synthesized 1D (C_4_N_2_H_14_)SnBr_4_ showed no PL and underwent a structural transformation under UV excitation to 0D (C_4_N_2_H_14_Br)_4_SnBr_6_ with a bright yellow emission. The 0D (C_4_N_2_H_14_Br)_4_SnBr_6_ showed excellent optical properties with a near‐unity PLQY.^[^ ^131^ ^]^ 0D Sn‐based perovskites possessed superior photostability in the ambient, because the PL centers were protected completely by the organic shells.^[^ ^132^ ^]^ All‐inorganic 0D Cs_4_SnBr_6_ perovskites with a PL emission at 540 nm and a PLQY of 15 ± 5% were synthesized. And a series of 0D Cs_4‐_ *_x_*A*_x_*Sn(Br_1‐_ *_y_*I*_y_*)_6_ (A = K^+^, Cs^+^) materials were also prepared and manifested the emission wavelength from 500 to 620 nm. As illustrated in Figure 6i, the PL mechanism of full inorganic 0D Sn‐based halide perovskites is ascribed to self‐trapping exciton emission. And the insets demonstrate that the HOMO (valence band) consists of Sn 5p and Br 5p orbitals, and LUMO (conduction band) consists of Sn 5s and Br 5p orbitals.^[^ ^133^ ^]^ In addition to the low‐dimensional Sn‐based halide perovskites, there are some nonperovskite structure Sn‐based halide materials reported. Morad et al. presented highly emissive single crystals of 0D disphenoidal Bmpip_2_SnBr_4_ and Bmpip_2_SnI_4_ (Bmpip = 1‐butyl‐1‐methylpiperidinium; C_20_H_44_N_2_) with emission peaks at 666 and 730 nm, respectively. In addition, Bmpip_2_SnBr_4_ also displayed intense emission under X‐ray excitation, which was comparable with the commercial inorganic X‐ray scintillator.^[^ ^134^ ^]^ Recently, Kanatzidis’ group synthesized 1D (DAO)Sn_2_I_6_ (DAO: 1,8‐octyldiammonium) single crystals and thin films. (DAO)Sn_2_I_6_ thin films with a PLQY of 36% demonstrated superior water stability, which could resist water for more than 15 h.^[^ ^135^ ^]^

The above discussion suggests that Sn‐based halide perovskites have made great advances in solar cells, light‐emitting devices, and X‐ray photodetectors over the past several years. As for Sn‐based perovskite light‐emitting materials, ultrabroad band PL emission with high PLQY is suitable for white‐light LED application. However, narrowband PL emission is not satisfactory. Instability and low PLQY are two significantly challenging issues. Notably, Sn‐based halide perovskites have a great potential in infrared light emission, which deserves more attention. In order to improve the stability and PLQY of Sn‐based halide perovskites, some effective strategies can be utilized, such as utilizing reducing agents H_3_PO_2_, SnF_2_, hydrazine vapor, and naphthol sulfonic salt, which have displayed obvious effects in Sn‐based halide perovskite solar cells.^[^ ^122^, ^136^ ^]^ Among the above reducing agents, H_3_PO_2_ and naphthol sulfonic salt have been proven to be effective in Sn‐based perovskite light‐emitting devices.^[^ ^136a,c^ ^]^ In order to better suppress the oxidation of Sn^2+^, it is necessary to clarify the oxidation pathway, which needs more efforts. If the stability problem of Sn(II) is solved, Sn(II) will be one of the most potential elements to substitute lead in the perovskite system due to its similar properties to Pb.

### 4.2 Ge‐Based Halide Perovskites

Germanium (Ge) is in the same group of lead (Pb) and tin (Sn) and has less toxicity than Pb, but more toxic than Sn. It is also considered as a potential candidate. However, Ge^2+^ is prone to be oxidized to Ge^4+^ owing to the active 4s lone pair electrons. There are quite a few reports about Ge‐based halide perovskites. Mhaisalkar's group reported Ge‐based halide perovskites, including three AGeI_3_ (A = Cs^+^, MA^+^, FA^+^) materials with *R*3*m* space group symmetry. The bandgaps of AGeI_3_ were 1.63, 2.0, and 2.35 eV for CsGeI_3_, MAGeI_3_, and FAGeI_3_.^[^ ^113^ ^]^ Mitzi synthesized single crystals of 2D (C_4_H_9_NH_3_)_2_GeI_4_ and studied the PL properties. The (C_4_H_9_NH_3_)_2_GeI_4_ had a weak emission peak at 690 nm with a FWHM of 180 nm.^[^ ^137^ ^]^ The 2D layered (PEA)_2_Ge_1‐_ *_x_*Sn*_x_*I_4_ (*x* ≤ 0.5) with direct bandgap showed emission peaks at wavelengths of 613 (*x* = 0), 628 (*x* = 0.125), 642 (*x* = 0.25), and 655 nm (*x* = 0.5) at room temperature. With the increase of Sn, the emission FWHM decreases possibly due to a more pronounced distortion of [GeI_6_]^4−^ octahedra than [SnI_6_]^4−^ octahedra. Time‐resolved PL suggested that there were two processes: charge‐carrier trapping for short‐lifetime and exciton recombination for long‐lifetime.^[^ ^138^ ^]^ Sadhanala's group presented Ge‐based and Sn‐based halide solid solutions and demonstrated the emission wavelength from 640 to 945 nm for the Ge content from 100% to 0.^[^ ^139^ ^]^ Single crystals of disphenoidal 0D Bmpip_2_GeBr_4_ were prepared with a red emission of 670 nm and a large Stokes shift of 330 nm.^[^ ^134^ ^]^

Recently, Yang's group identified 23 lead‐free halide perovskites for light‐emitting diodes via high‐throughput computational design, including (MA)_2_GeBr_4_, (MA)_2_GeI_4_, (AD)_2_GeI_4_ (AD = (CH_2_)_2_NH_2_)), which provides a new direction to develop new‐type lead‐free halide perovskites.^[^ ^140^ ^]^ Researchers can seek for suitable synthesis method to prepare the proposed Ge‐based halide perovskites and study relevant optical and optoelectrical properties. Combining theory with experiment will greatly promote the development of lead‐free perovskite optoelectronics.

### 4.3 Bi‐Based Halide Perovskites

**Figure 7:**
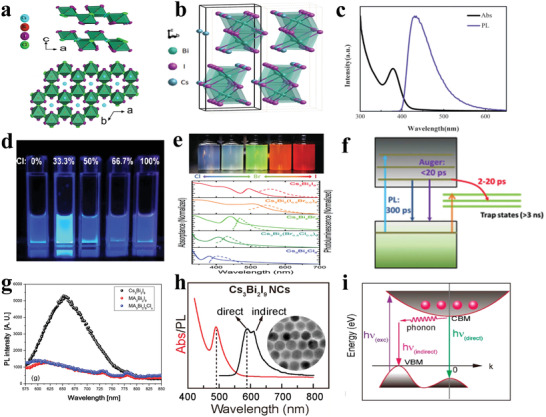
a) 2D crystal structure of Cs_3_Bi_2_X_9_ was displayed viewed down the b‐axis and c‐axis. Reproduced with permission.^[^ ^154^ ^]^ Copyright 2019, American Chemical Society. b) 0D Bi‐based halide perovskite structure consists of dioctahedral face‐sharing (Bi_2_X_9_)^3−^ clusters with hexagonal phase. Reproduced with permission.^[^ ^147^ ^]^ Copyright 2015, Wiley‐VCH. c) Absorption and PL spectra of MA_3_Bi_2_Br_9_ nanocrystals. Reproduced with permission.^[^ ^143^ ^]^ Copyright 2016, Wiley‐VCH. d) Photographs of MA_3_Bi_2_Br_9_ nanocrystal solutions passivated with different amount of Cl^−^ under a 325 nm UV lamp excitation. Reproduced with permission.^[^ ^59^ ^]^ Copyright 2018, American Chemical Society. e) Photographs (top) and steady‐state absorption and PL spectra (bottom) of colloidal Cs_3_Bi_2_X_9_ (X = Cl, Cl_0.5_Br_0.5_, Br, Br_0.5_I_0.5_, I) nanocrystals. f) Excited dynamics model of Cs_3_Bi_2_Br_9_ nanocrystals via combining time‐resolved PL and transient absorption. Reproduced with permission.^[^ ^144^ ^]^ Copyright 2017, Wiley‐VCH. g) PL spectra for the Cs_3_Bi_2_I_9_, MA_3_Bi_2_I_9_ and MA_3_Bi_2_I_9_Cl*_x_* thin films. Reproduced with permission.^[^ ^147^ ^]^ Copyright 2015, Wiley‐VCH. h) Normalized absorption and PL spectra of Cs_3_Bi_2_I_9_ colloidal nanocrystals. i) Proposed recombination process in Cs_3_Bi_2_I_9_ nanocrystals. Reproduced with permission.^[^ ^152^ ^]^ Copyright 2017, American Chemical Society.

Lead‐based halide perovskites present superior optoelectrical characteristics, which are strongly relevant to the 6s^2^6p^0^ electronic configuration of Pb^2+^. However, only three stable cations have the 6s^2^6p^0^ electronic configuration, namely, Tl^+^, Pb^2+^, and Bi^3+^. Among these, only Bi^3+^ ions have relatively low toxicity, which promotes Bi‐based halide perovskites to be a valid alternative. Additionally, Bi‐based halide perovskites were identified as defect‐tolerant semiconductors via computational screening.^[^ ^141^ ^]^ Among Bi‐based halide perovskites, a common formula is A_3_Bi_2_X_9_, where A stands for Cs^+^, Rb^+^ or MA^+^ and X refers to halogen anion Cl^−^, Br^−^, and I^−^. At the molecular level, there are different dimensionalities for A_3_Bi_2_X_9_ according to various connection types between the adjacent Bi‐based halide octahedra. The 2D crystal structure of Cs_3_Bi_2_X_9_ was viewed down the b‐axis and c‐axis as shown in **Figure** **7**a. The 0D Bi‐based halide perovskite structure consists of dioctahedral face‐sharing (Bi_2_X_9_)^3−^ clusters with hexagonal phase as can be seen from Figure 7b. The dimensionality of Bi‐based halide perovskites can also be ascertained via choosing suitable organic cations in A site, such as 1D (C_6_H_13_N)_2_BiI_5_ with a zigzag chain structure and 2D (TMP)_1.5_[Bi_2_I_7_Cl_2_] with a honeycomb shape (TMP = *N,N,N′,N′*‐tetramethylpiperazine).^[^ ^142^ ^]^ In recent years, there is a great number of Bi‐based halide perovskites reported, we summarize the Bi‐based halide perovskites, mainly concentrate on light generation.

#### 4.3.1 2D Bi‐Based Halide Perovskites

Tang's group reported that single crystals and nanocrystals of MA_3_Bi_2_Br_9_ were synthesized by evaporating the solvent of the saturated solution and collaborative solvent ligand‐assisted reprecipitation (Co‐LARP), respectively. The MA_3_Bi_2_Br_9_ nanocrystals with an average diameter of 3.05 nm showed an emission peak around 423 nm with FWHM of 62 nm as shown in Figure 7c, manifesting a huge blue‐shift (compared to the single crystals) due to quantum confinement effect.^[^ ^143^ ^]^ Afterwards, the optical properties of Cl^−^ anion passivated MA_3_Bi_2_Br_9_ nanocrystals were studied. As shown in Figure 7d, a significant boost of PLQY (54.1%) was observed where the surface defects were suppressed effectively. Time‐resolved PL measurements with a monoexponential fitting and temperature‐related PL showing larger exciton binding energy verified the superior passivation effect.^[^ ^59^ ^]^ In general, the all‐inorganic halide perovskites have better stability than the organic–inorganic hybrid halide perovskites, because organic cations are vulnerable to decomposition. Correspondingly, all‐inorganic Cs_3_Bi_2_X_9_ nanocrystals were also prepared by Tang's group and Han's group.^[^ ^58^, ^144^ ^]^ Tang's group utilized ethanol as the main solvent to prepare Cs_3_Bi_2_X_9_ nanocrystals, which displayed a blue emission at 410 nm with a PLQY of 19.4% and a superior stability. Notably, the Cs_3_Bi_2_Br_9_ nanocrystals displayed a significant PL enhancement after some amount of water was added into the ethanol solution containing nanocrystals. The phenomenon was ascribed to the passivation effect of BiOBr.^[^ ^58^ ^]^ The effect of coordinating H_2_O in lead‐free halide perovskites was also studied. In addition to decomposition, water also influenced the crystallization of perovskites. Zhang et al. reported that suitable amount of water improved the PLQY of perovskites and changed the size and shape of perovskite nanocrystals.^[^ ^145^ ^]^ Han's group synthesized Cs_3_Bi_2_X_9_ nanocrystals using DMSO as solvent and isopropanol as the antisolvent via a simple and scalable synthesis method, exhibiting a tunable emission wavelength though composition adjustment as shown in Figure 7e.^[^ ^144^ ^]^ Besides, Han's group carried out the transient absorption measurement and the time‐resolve PL in the Cs_3_Bi_2_Br_9_ nanocrystals, which provided an extensive comprehension on the charge‐carrier dynamics involving both radiative and non‐radiative processes.

Excited state dynamics of ligand‐free Cs_3_Bi_2_Br_9_ nanocrystals suggested that Auger recombination and charge transfer from excited states to trapping states took place in 2–20 ps, intrinsic radiative combination happened at 300 ps, and the decay of the long‐lived trapping states occurred at more than 3 ns as shown in Figure 7f. Excited state dynamic process is responsible for the low PLQY of the ligand‐free Cs_3_Bi_2_Br_9_ nanocrystals. Additionally, the ultrafast component greatly decreased in the OA capped Cs_3_Bi_2_Br_9_ NCs, which suggested that Cs_3_Bi_2_Br_9_ nanocrystals could be passivated effectively via the use of oleic acid as the surfactant.^[^ ^144^ ^]^

The broad PL of single crystals of 2D Rb_3_Bi_2_I_9_ and Cs_3_Bi_2_I_9_ was observed at room temperature, which was ascribed to inducing small polarons and resulting in trapping of excitons by the lattice. Moreover, the similar effective phonon energy was obtained by temperature‐dependence PL and Raman spectra. Rb_3_Bi_2_I_9_ and Cs_3_Bi_2_I_9_ presented high resistivity and high photoresponse under the laser photoexcitation, which demonstrated their potentials in detector applications.^[^ ^146^ ^]^

#### 4.3.2 0D Bi‐Based Halide Perovskites

In 2015, thin films of 0D Bi‐based halide perovskite were synthesized by Park et al. 0D Cs_3_Bi_2_I_9_ with larger exciton binding energy of 270 meV displayed strong PL (Figure 7g).^[^ ^147^ ^]^ The nanocrystals had a hexagonal *P*63*mmc* space group and exhibited broad PL emission, which was attributed to free excitons and defect emission.^[^ ^148^ ^]^ Strikingly, two peaks in the PL spectra of Cs_3_Bi_2_I_9_ nanocrystals were observed at room temperature, which were centered at 580 nm (2.14 eV) and 605 nm (2.05 eV) (Figure 7h. The inset shows the morphology).^[^ ^149^ ^]^ Moreover, early investigations revealed that the direct bandgap transition and indirect bandgap transition had vastly different behaviors with the increasing temperature in theory. Generally, the radiative recombination via direct bandgap transition decreases with rising temperature due to the thermal quenching effect.^[^ ^150^ ^]^ On the contrary, the radiative recombination through indirect bandgap transition can be boosted with elevated temperature, which is ascribed to the additional momentum compensation from phonons to obey the momentum conservation.^[^ ^151^ ^]^ The two different phenomena were indeed observed. Furthermore, DFT calculations shown the presence of direct and indirect bandgaps. In addition, the relevant PL mechanism (Figure 7i) is that the electrons in the valence band jump to the conduction band under excitation involving the direct bandgap transition, as shown in the absorption spectra. With the help of phonons, the indirect bandgap transition and direct bandgap transition occurred corresponding to the 605 and 580 nm emission.^[^ ^152^ ^]^

Intriguingly, mixed halides contribute to the molecular dimensionality transformation from 0D Cs_3_Bi_2_I_9_ to 2D Cs_3_Bi_2_I_6_Cl_3_, and this phenomenon highlights the importance of interlayer interactions in the defect perovskite family.^[^ ^153^ ^]^ Song's group reported a new kind of Bi‐based perovskite, namely, Rb_7_Bi_3_Cl_16_ in the form of single crystals and nanocrystals. Rb_7_Bi_3_Cl_16_ had a special crystal structure, which displayed as a 0D cluster composed of two kinds of octahedra with different distortions. Rb_7_Bi_3_Cl_16_ nanocrystals with an average diameter of 1.85 nm exhibited a blue emission at 437 nm with PLQY of 28.43% and FWHM of 96 nm, providing a direction to develop new lead‐free halide perovskites.^[^ ^79^ ^]^

Compared with Sn‐based and Ge‐based halide perovskites, Bi‐based halide perovskites possess superior stability. Remarkably, Bi‐based halide perovskites belong to defect‐tolerant perovskites. In terms of the currently developed Bi‐based halide perovskites, most of them are low‐dimension perovskites. As light‐emitting materials, low‐dimension perovskites have larger exciton binding energies, which is beneficial for exciton recombination to obtain higher PLQY. However, low‐dimension perovskites generally possess inferior carrier transport, which is detrimental in optoelectrical applications. There are several challenges for Bi‐based halide perovskite materials. First, their structure–property relationships and underlying mechanisms are extremely unclear. Second, Bi‐based halide perovskites with direct bandgap are scarce, which can be obtained via experimental tools and theoretical calculations. Third, the thin film quality of Bi‐based halide perovskites needs to be improved. Some common methods can be attempted, including optimizing the precursor solution, solvent‐engineering to decrease the volatility of the precursor solutions and their rapid crystallization.

### 4.4 Sb‐Based Halide Perovskites

**Figure 8:**
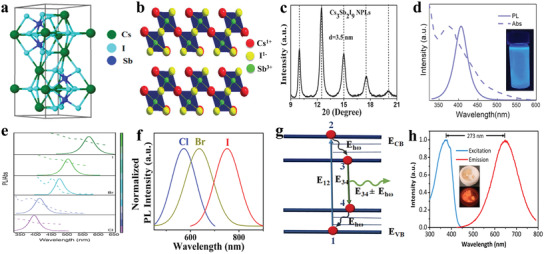
a) Crystal structure of 0D Cs_3_Sb_2_I_9_. Reproduced with permission.^[^ ^157^ ^]^ Copyright 2016, American Chemical Society. b) Crystal structure of 2D Cs_3_Sb_2_I_9_. c) XRD of Cs_3_Sb_2_I_9_ nanoplates with periodic angle intervals. Reproduced with permission.^[^ ^96^ ^]^ Copyright 2017, Wiley‐VCH. d) UV−vis absorption and PL spectra of Cs_3_Sb_2_Br_9_ nanocrystals. Inset: image of a colloidal Cs_3_Sb_2_Br_9_ QD solution under the excitation of 365 nm. e) Absorption and PL spectra of Cs_3_Sb_2_X_9_ NCs via halide substitution. Reproduced with permission.^[^ ^80^ ^]^ Copyright 2017, American Chemical Society. f) Normalized PL spectra of Cs_3_Sb_2_X_9_ (X = Cl, Br, I) thin films. g) Broad emission model of Cs_3_Sb_2_X_9_ (X = Cl, Br, I) thin films with nonradiative recombination due to phonons. Reproduced with permission.^[^ ^156^ ^]^ Copyright 2019, American Chemical Society. h) Excitation and emission spectra of bulk crystals (Ph_4_P)_2_SbCl_5_. Inset: typical optical images of bulk (Ph_4_P)_2_SbCl_5_ crystals without and with 365 nm UV light excitation. Reproduced with permission.^[^ ^82^ ^]^ Copyright 2018, American Chemical Society.

It is reasonable to substitute Pb^2+^ ions in perovskite structure by antimony (Sb) apart from Sn, Ge, and Bi elements. Structurally, stable A_3_Sb_2_X_9_ can be derived from 2/3 occupancy of the B sites in the A_3_B_3_X_9_ perovskite, which can be considered as “defect perovskites.” There are two perovskite phases, including 0D dimer form (space group P63/mmc, No. 194) and 2D layered form (space group *P*3*m*1, No.164), which can be formed depending on the synthesis conditions.^[^ ^99b^ ^]^ The 0D dimer phase perovskites consist of dioctahedral face‐sharing (Sb_2_X_9_)^3−^ clusters, which can be synthesized via a low‐temperature solution process (**Figure** **8**a). The 2D perovskites consist of corrugated layer structure with partially corner‐sharing MX_6_ octahedra, which are usually thermodynamically unstable (Figure 8b). The change in dimensionality has a huge influence on the optical and electronic properties of perovskite materials.

#### 4.4.1 2D Sb‐Based Halide Perovskites

2D colloidal Cs_3_Sb_2_I_9_ and Rb_3_Sb_2_I_9_ nanocrystals synthesized by the hot injection method were studied.^[^ ^96^ ^]^ The Cs_3_Sb_2_I_9_ nanoplates and Cs_3_Sb_2_I_9_ nanorods can be derived from different reaction temperatures. As shown in Figure 8c, the Cs_3_Sb_2_I_9_ nanoplates display an XRD pattern with periodic property, which confirms that the materials belong to the 2D structure. The PL lifetime measurements provided some valuable information about Cs_3_Sb_2_I_9_ nanoplates and nanorods, wherein the nonradiative decay with lifetimes of about 1 ns decreased the PL efficiency to about 5%.^[^ ^96^ ^]^ The underlying reason is the deep defect levels in Cs_3_Sb_2_I_9_ possibly originated from both localization and reduction of the spin–orbit splitting of Sb 5p‐orbital.^[^ ^99b^ ^]^ The single crystals and quantum dots of Cs_3_Sb_2_Br_9_ were prepared.^[^ ^80^ ^]^ <111>‐stacked 2D layered Cs_3_Sb_2_Br_9_ nanocrystals exhibited remarkable optical properties, including blue emission at 410 nm, PLQY of 46%, FWHM of 41 nm and tunable emission wavelength from 370 to 560 nm via anion exchange reactions (Figure 8d,e). The excellent optical characteristics were mainly attributed to two aspects. On the one hand, Br‐rich surface passivated the defects of Cs_3_Sb_2_Br_9_ nanocrystals; on the other hand, larger exciton binding energy of 530 meV demonstrated more efficient exciton radiative recombination.^[^ ^80^ ^]^ Zuo et al. reported that (NH_4_)_3_Sb_2_I*_x_*Br_9‐_ *_x_* single crystals and thin films were synthesized by antisolvent vapor‐assisted crystallization method (AVC) and spin‐coating method, respectively. The (NH_4_)_3_Sb_2_I_9_ single crystals demonstrated an absorption onset at 645 nm and a PL emission at 639 nm. Whereas the uniform and compact thin films of (NH_4_)_3_Sb_2_I_9_ presented an absorption onset at 558 nm.^[^ ^155^ ^]^ The 87 nm blue shift phenomenon was also observed in lead halide perovskite single crystals and thin films, which indicates that the excitons dissociate to free charges at room temperature. Recently, Chu's team presented 2D layered Cs_3_Sb_2_I_9_ thin films for LEDs. 2D layered Cs_3_Sb_2_I_9_ thin films with direct bandgap showed an emission peak at 750 nm with a FWHM of 120 nm and tunable emission wavelengths as shown in Figure 8f. A model explained these broad emissions, where the interactions between radiative transitions and phonons are presented, as shown in Figure 8g. Absorption transition occurs between energy levels 1 and 2, and the emission transition happens from energy 3 to energy 4 after lattice interaction with phonons.^[^ ^156^ ^]^

#### 4.4.2 0D Sb‐Based Halide Perovskites

In addition to the 2D Sb‐based halide perovskites, 0D Sb‐based halide perovskites are also widely studied. The fabricated 0D (CH_3_NH_3_)_3_Sb_2_I_9_ thin films showed a PL emission at 1.58 eV.^[^ ^157^ ^]^ The 0D mixed Sb‐ and Bi‐based perovskite single crystals of (C_8_NH_12_)_4_Bi_0.57_Sb_0.43_Br_7_∙H_2_O synthesized by a solution process demonstrated an ultrabroad band emission spectrum from 400 to 850 nm, which was attributed to the combination of free excitons and self‐trapped excitons.^[^ ^78^ ^]^ Recently, 0D Sb‐based halide hybrid materials with pyramidal SbCl_5_ structure also show superior optical characteristics. The single crystals of 0D (C_9_NH_20_)_2_SbCl_5_ show a PL emission at 590 nm, an extremely high PLQY of close to unity and a wide FWHM of 119 nm, which is attributed to exciton self‐trapping.^[^ ^132^ ^]^ As shown in Figure 8h, 0D (Ph_4_P)_2_SbCl_5_ bulk single crystals with high thermostability and photostability also demonstrated superior optical characteristics including a PLQY of 87%, a broad red emission at 648 nm and a phosphorescent lifetime of 4.57 ± 0.09 µs. In addition, theory calculations explained that a stronger excited state distortion from a shorter Sb—Cl bond led to a larger Stoke shift of (Ph_4_P)_2_SbCl_5_ (273 nm) than (C_9_NH_20_)_2_SbCl_5_ (210 nm).^[^ ^82^ ^]^

Currently reported Sb‐based halide perovskites are summarized. There are some prospects and challenges for Sb‐based halide perovskites. 0D Sb‐based halides bulk crystals with high PLQY and larger Stoke shift demonstrate outstanding potentials in WLED lighting and X‐ray scintillator. Recently, Ma et al. adopted a modified recrystallization method to obtain a PLQY of 51.2%, which gives us an idea to optimize the synthesized lead‐free halide perovskites to gain superior performance.^[^ ^66b^ ^]^ Above optimization needs us to understand the source of the defects. The challenges lie in the following aspects. On the one hand, Sb‐based halide perovskites serve as “defect perovskites,” and there are indeed high contents of deep‐level defects in the A_3_B_2_X_9_ structure. On the other hand, Sb‐based halide perovskites still have the problem of poor morphology. Seeking suitable synthesis methods to obtain high‐quality Sb‐based halide thin films is necessary.^[^ ^156^ ^]^

### 4.5 Halide Double Perovskites

**Figure 9:**
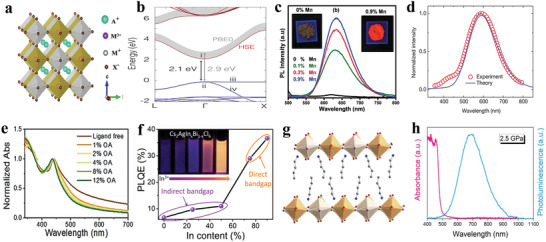
a) Crystal structure of halide double perovskites. Reproduced with permission.^[^ ^62^ ^]^ Copyright 2018, Wiley‐VCH. b) The band structure and bandgaps of Cs_2_AgInCl_6_ calculated by HSE hybrid functional (blue and red lines) and PBE0 functional (shaded area). The maximum of valence band is set to the energy zero. Reproduced with permission.^[^ ^161^ ^]^ Copyright 2018, Wiley‐VCH. c) PL spectra of Mn^2+^ doped Cs_2_AgInCl_6_ samples with different contents under 340 nm excitation. Inset: images of luminescence of powder under UV excitation. Reproduced with permission.^[^ ^163^ ^]^ Copyright 2018, Royal Society of Chemistry. d) Calculated photoluminescence spectrum and experimental photoluminescence spectrum of Cs_2_AgInCl_6_. Reproduced with permission.^[^ ^60^ ^]^ Copyright 2018, Springer Nature. e) Steady‐state absorption spectra of ligand‐free and OA‐capped Cs_2_AgBiBr_6_ nanocrystals. Reproduced with permission.^[^ ^62^ ^]^ Copyright 2018, Wiley‐VCH. f) Schematic representation of the change of bandgap type with different In^3+^/Bi^3+^ ratio and corresponding PLQY. Inset: PL image of ligand‐free Cs_2_AgIn_x_Bi_1−x_Cl_6_ (x = 0, 0.25, 0.5, 0.75, and 0.9) nanocrystals under UV light of 365 nm. Reproduced with permission.^[^ ^24^ ^]^ Copyright 2018, American Chemical Society. g) Single‐crystal X‐ray structures (298 K) of the (001) layered double perovskites (BA)_4_AgBiBr_8_. Orange, white, turquoise, brown, blue, and gray spheres represent Bi, Ag, Cs, Br, N, and C atoms, respectively and H atom is omitted for clarity. Reproduced with permission.^[^ ^170a^ ^]^ Copyright 2018, American Chemical Society. h) Absorption (pink) and emission (blue) spectra for (BA)_4_AgBiBr_8_ at 2.5 GPa. Reproduced with permission.^[^ ^171^ ^]^ Copyright 2019, Wiley‐VCH.

Recently, the halide double perovskites with a formula of A_2_M^+^M^3+^X_6_ (A = Cs^+^, MA^+^; M^+^ = Ag^+^, Na^+^, In^+^; M^3+^ = In^3+^, Bi^3+^, Sb^3+^; X = Cl^−^, Br^−^, I^−^) and the possible element substitutes are presented in Figure 1. Halide double perovskites have gained widespread interest as candidates for lead‐free halide perovskite materials, owing to superior stability against light, heat, and moisture. In fact, the double perovskite structure is also named elpasolite, which is known for its ferroelectric properties since the 1960s. However, the photoelectronic properties of halide double perovskites were only recently investigated. Typically, halide double perovskites present 3D structure at the molecular level, wherein [M^+^X_6_] and [M^3+^X_6_] corner‐sharing octahedra are alternately arranged (**Figure** **9**a). There are a large number of halide double perovskite materials, including single crystals, thin films, and nanocrystals for different photoelectronic applications such as light‐emitting diodes, solar cells, photodetectors, X‐ray detectors.^[^ ^60^, ^158^ ^]^

In 2016, many research groups reported the synthesis of Cs_2_AgBiX_6_ (X = Cl, Br, I) double perovskite materials via solid‐state reaction and solution process.^[^ ^76^, ^159^ ^]^ The cubic Cs_2_AgBiBr_6_ powders with a space group *Fm*‐3*m* were obtained by Karunadasa's group, which displayed a weak PL emission at 1.87 eV at room temperature.^[^ ^76^ ^]^ Greul et al. reported that the Cs_2_AgBiBr_6_ thin films had a broad PL with low PLQY, which was likely attributed to the nonradiative defect effect.^[^ ^88^ ^]^

The band structure calculations and optical measurements suggested that Cs_2_AgBiBr_6_ and Cs_2_AgBiCl_6_ belonged to indirect bandgap semiconductors, which are not ideal for solar cells and light‐emitting applications.^[^ ^160^ ^]^ Notably, a novel halide double perovskite Cs_2_AgInCl_6_ with direct bandgap was demonstrated by Volonakis et al. in 2017.^[^ ^161^ ^]^ In Figure 9b, density functional theory (DFT) indicated that the conduction band of Cs_2_AgInCl_6_ originated from Cl‐3p and In‐5s/Ag‐5s states, and the valence band was attributed to Cl‐3p and In‐4d/Ag‐4d states. Moreover, Cs_2_AgInCl_6_ powders demonstrated a PL emission at 608 nm with FWHM of 120 nm.^[^ ^161^ ^]^ Simultaneously, the investigation of bandgap engineering was also carried out in halide double perovskites.^[^ ^35^ ^]^ In the trivalent metals alloyed Cs_2_AgBiBr_6_ system, the bandgap of Cs_2_AgBiBr_6_ can be enlarged by increasing the contents of In^3+^ in the Cs_2_AgBi_1‐_ *_x_*In*_x_*Br_6_, whereas the bandgap decreased via increasing the contents of Sb^3+^ in the Cs_2_AgBi_1‐_ *_x_*Sb*_x_*Br_6_.^[^ ^162^ ^]^

Afterwards, Mn^2+^ doped halide double perovskites were presented. Mn^2+^ doped Cs_2_AgInCl_6_ double perovskite emitted red light under UV excitation, as shown in Figure 9c.^[^ ^163^ ^]^ It is worth mentioning that Cs_2_Ag_0.6_Na_0.4_InCl_6_:0.04Bi^3+^ synthesized by hydrothermal method demonstrated stable emission of warm white light (460–700 nm) with PLQY of 86±5%.^[^ ^60^ ^]^ The broad‐band emission of Cs_2_AgInCl_6_ originates from self‐trapped excitons (STEs) as shown in Figure 9d, which was confirmed experimentally and theoretically. The excitation spectra presented similar shapes and features under the emission from 460 to 700 nm in Cs_2_Ag_0.6_Na_0.4_InCl_6_:0.04Bi^3+^, suggesting that white emission is originated from the same excited state. Moreover, the PLQY was independent of the excitation power, meaning that the white emission was not attributed to the permanent defects. In addition, the transient absorption spectra further directly verified the presence of STEs. These experimental results were consistent with the calculation results of exciton self‐trapping time in theory. In this system, the roles of Na^+^ are to break the parity­forbidden transition and reduce electronic dimensionality, which results in efficient white emission via radiative recombination of STEs. And the roles of Bi^3+^ are considered to improve the crystal perfection and localized exciton.^[^ ^60^ ^]^ Subsequently, alkali‐metal ions Li^+^ and K^+^ have also been introduced to the Cs_2_AgInCl_6_ NCs, modifying the white‐light emission.^[^ ^164^ ^]^

Over a span of less than two years, a large number of colloidal nanocrystals of halide double perovskite has been synthesized.^[^ ^84^, ^165^ ^]^ The ligand‐free Cs_2_AgBiBr_6_ nanocrystals with an average diameter of 5.0 nm demonstrated an exciton absorption peak at 440 nm with a long absorption tail up to 700 nm. The absorption tail means the existence of a transition involving sub‐bandgap states, which may attribute to the defect states of nanocrystals. As shown in Figure 9e, we can observe that the tail phenomenon is suppressed in the oleic acid capped halide double perovskite nanocrystals. The phenomenon suggested that oleic acid can serve as the surfactant to passivate the defects in Cs_2_AgBiCl_6_ nanocrystals, thus increasing PLQY.^[^ ^62^ ^]^ Manna's group first reported colloidal Cs_2_AgInCl_6_ nanocrystals and Mn^2+^ doped Cs_2_AgInCl_6_ nanocrystals with good size distributions. Cs_2_AgInCl_6_ nanocrystals displayed a broad white‐emitting PL with a PLQY of ≈1.6 ± 1%, and a higher PLQY of ≈16 ± 4% was obtained in orange‐emitting Mn^2+^ doped Cs_2_AgInCl_6_ nanocrystals.^[^ ^66c^ ^]^ In addition to Mn^2+^ doping, Bi^3+^ and lanthanide ions (Tb^3+^, Yb^3+^, Er^3+^) doped Cs_2_AgInX_6_ nanocrystals have also been synthesized and Bi^3+^ doped Cs_2_AgIn_1‐x_Bi_x_X_6_ emitted orange light.^[^ ^84b^ ^]^ Subsequently, Xia's group reported that Tb^3+^ doped Cs_2_AgIn_1‐x_Bi_x_X_6_ nanocrystals. Their emissions could be adjusted from green light to orange light, which was ascribed to the energy transfer channel from self‐trapped excitons to Tb^3+^ ions.^[^ ^166^ ^]^ Upon Yb^3+^ and Er^3+^ doping, the emissions of 996 nm for Yb^3+^ and 1537 nm for Er^3+^ dopants were observed in Cs_2_AgInCl_6_ nanocrystals. The introduction of these dopants expands the emission range and facilitates relevant luminescence applications, including optical communication, plant growth, and night‐vision devices.^[^ ^84^, ^167^ ^]^

Interestingly, Han's group reported Cs_2_AgIn*_x_*Bi_1−_ *_x_*Cl_6_ (*x* = 0, 0.25, 0.5, 0.75, and 0.9) nanocrystals with different bandgap types, including direct bandgap (*x* = 0.75, 0.9) and indirect bandgap (*x* = 0, 0.25, 0.5) as shown in Figure 9f. Halide double perovskite nanocrystals with different bandgap types manifested disparate optical features and charge carrier dynamics. Cs_2_AgIn_0.75_Bi_0.25_Cl_6_ and Cs_2_AgIn_0.9_Bi_0.1_Cl_6_ nanocrystals with direct bandgap demonstrated dual‐color emission (violet emission of 395 nm and orange emission of 570 nm), whereas Cs_2_AgIn*_x_*Bi_1−_ *_x_*Cl_6_ nanocrystals (*x* = 0, 0.25, 0.5) showed only one emission peak (400−410 nm). Femtosecond transient absorption measurements unveiled the essence of radiative and nonradiative processes with different bandgap types. The rapid component of GSB (ground‐state bleach) decay is attributed to the intrinsic sub‐bandgap trapping. And bleaching signal at long wavelengths originates from the sub‐bandgap trap‐state absorption and indirect bandgap transition. In Cs_2_AgIn_0.9_Bi_0.1_Cl_6_ nanocrystals, there is only a positive PIA (photoinduced absorption) decay signal observed.^[^ ^24^ ^]^

Recently, Cs_2_Ag*_x_*Na_1‐_ *_x_*In*_y_*Bi_1‐_ *_y_*Cl_6_ nanocrystals are under research spotlights, which were described by Manna's group and Tang's group successively. Manna et al. reported that Bi^3+^ doped Cs_2_Ag_0.4_Na_0.6_InCl_6_ nanocrystals synthesized by the hot injection method showed PLQY of 22%. Tang's group reported that Cs_2_Ag_0.17_Na_0.83_In_0.88_Bi_0.12_Cl_6_ nanocrystals prepared by room temperature recrystallization process displayed a broad emission with PL peak of 557 nm and PLQY of 64%. Meanwhile, the color temperature could be adjusted by changing Na^+^ and Bi^3+^ doping contents.^[^ ^168^ ^]^ Han's group synthesized cubic Cs_2_NaInCl_6_ nanocrystals with a direct bandgap and Ag^+^ doped Cs_2_NaInCl_6_ nanocrystals. The research demonstrated that Ag^+^ doped Cs_2_NaInCl_6_ nanocrystals exhibited a broad PL emission peak (centered at 535 nm in a range of 400−750 nm) originating from STEs. That was directly verified by the transient absorption measurements. Furthermore, Ag^+^ doping also improved the stability in comparison with the undoped materials.^[^ ^85^ ^]^ Moreover, Mn^2+^ doped Cs_2_NaIn*_x_*Bi_1−_ *_x_*Cl_6_ (0 ≤ *x* ≤ 1) NCs were also synthesized, and the emission color could be adjusted from yellow to orange‐red, with a PLQY of 44.6%.^[^ ^169^ ^]^

The above‐mentioned halide double perovskites are 3D perovskite structure. Similarly, there are several investigations for 2D halide double perovskites.^[^ ^170^ ^]^ Mitzi's team prepared 2D halide double perovskites [AE2T]_2_AgBiI_8_ (AE2T = 5,5’‐diylbis(aminoethyl)‐[2,2’‐bithiophene]) which was predicted to be a semiconductor with a direct bandgap. However, [AE2T]_2_AgBiI_8_ was barely luminescent at room temperature even at 78 K temperature.^[^ ^170b^ ^]^ For (BA)_4_AgBiBr_8_ (BA = CH_3_(CH_2_)_3_NH_3_) ), as shown in Figure 9g, it displayed a weak and slightly broadened PL at 20 K, which was quenched rapidly upon warming. However, (BA)_4_AgBiBr_8_ demonstrated obvious PL under the condition of the pressure of 2.5 GPa as shown in Figure 9h.^[^ ^170^, ^171^ ^]^ A unique <111>‐oriented mixed metal layered perovskite Cs_4_CuSb_2_Cl_12_ with a direct bandgap was synthesized by Vargas et al., which provides us a new strategy for material design with the various substitutions in the M^+^ and M^3+^ sites.^[^ ^172^ ^]^ Besides, 2D vacancy‐ordered double perovskites Cs_4_MnBi_2_Cl_12_, Cs_4_CdBi_2_Cl_12_ and Cs_4_Cd_1‐x_Mn_x_Bi_2_Cl_12_ were reported continuously, enriching the family of lead‐free halide perovskites.^[^ ^173^ ^]^

Halide double perovskites have the flexibility for various compositional adjustments. In the future, the research for lead‐free halide double perovskites may direct to the following aspects. Conversion of bandgap type and break of parity‐forbidden transition in halide double perovskites by doping or alloying ions deserve further research. In addition, the high‐quality thin films can be further explored via different vapor deposition process. Moreover, in order to transport carriers effectively, high electronic dimensionality should be guaranteed.

### 4.6 Cu and Other Metal‐Based Halide Perovskites

**Figure 10:**
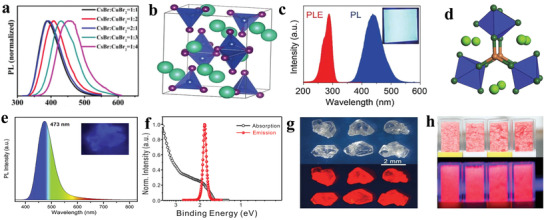
a) PL spectra of Cs_2_CuBr_4_ nanocrystals by altering the stoichiometric ratio of CsBr and CuBr_2_ under a 365nm UV lamp. Reproduced with permission.^[^ ^175^ ^]^ Copyright 2018, Royal Society of Chemistry. b) Crystal structure of Cs_3_Cu_2_I_5_, where blue corresponds to [Cu_2_I_5_]^3−^. c) PL and PLE spectra of the Cs_3_Cu_2_I_5_ thin film fabricated on a glass substrate. Inset: image of Cs_3_Cu_2_I_5_ thin film under the excitation of 245 nm monochromatic light. Reproduced with permission.^[^ ^61^ ^]^ Copyright 2019, Wiley‐VCH. d) Schematic of two 3‐coordinated monovalent Cu(I) ions connecting three [ScCl_6_]^3−^ octahedra to form a [Cu_2_(ScCl_6_)_3_]^7−^ cluster. Light‐green, dark‐green, and orange spheres represent Rb, Cl, and Cu atoms, respectively; Sc atoms are in the blue octahedra. e) PL spectrum of Rb_8_CuSc_3_Cl_18_. Inset: PL image of Rb_8_CuSc_3_Cl_18_. Reproduced with permission.^[^ ^178^ ^]^ Copyright 2019, Cell press. f) Absorption and PL spectra of the CsYbI_3_ nanocrystals. Reproduced with permission.^[^ ^180^ ^]^ Copyright 2019, Wiley‐VCH. g) Photographs of Cs_2_InBr_5_·H_2_O single crystals under ambient light (top) and UV light (bottom). Reproduced with permission.^[^ ^72^ ^]^ Copyright 2019, Wiley‐VCH. h) Images of (Pyrrolidinium)MnCl_3_ crystals under ambient light (top) and UV light (bottom). Reproduced with permission.^[^ ^185^ ^]^ Copyright 2019, Wiley‐VCH.

Currently, Cu‐based halide perovskites have received great attention for the abundance, low cost, and environmental friendliness of Cu element. In 2016, Mathews’ group first reported 2D Cu‐based perovskites MA_2_CuCl*_x_*Br_4‐_ *_x_* powders and films, which were synthesized by spontaneous crystallization and spin‐coating, respectively. These powders’ bandgaps varied with increasing Br contents.^[^ ^174^ ^]^ Afterwards, Cu‐based perovskite materials appeared widely in research. Yang et al. synthesized Cs_2_CuX_4_ (X = Cl, Br, Br/I) nanocrystals via an improved ligand‐assisted reprecipitation technique at room temperature. The PL wavelengths of Cs_2_CuX_4_ nanocrystals with blue‐green emitting could be adjusted via the molar ratio of the raw materials as shown in **Figure** **10**a.^[^ ^175^ ^]^ Notably, monovalent Cu(I)‐based materials were also intensively applied to light generation, but they do not belong to perovskite or perovskite analogy. As shown in Figure 10b, the unit consists of tetrahedrons instead of octahedra. These materials have excellent optical properties with environmental friendliness.

Saparov's group reported that the Cs_3_Cu_2_Br_5‐_ *_x_*I*_x_* phosphors have a maximum emission wavelength from 443 to 455 nm with near‐unity PLQY.^[^ ^176^ ^]^ Jun et al. prepared 0D Cs_3_Cu_2_I_5_ single crystals and thin films via vapor saturation of an antisolvent (VSA) and spin‐coating, respectively. A 5 mm Cs_3_Cu_2_I_5_ single crystal showed 445 nm PL emission with high PLQY of 90% and the greatly stable Cs_3_Cu_2_I_5_ thin films displayed 445 nm with high PLQY of 62% as shown in Figure 10c.^[^ ^61^ ^]^ Afterwards, corresponding nanocrystals were presented. Han's group reported 0D Cs_3_Cu_2_I_5_ nanocrystals and 1D CsCu_2_I_3_ nanorods synthesized by the hot injection approach under different temperatures. The 1D CsCu_2_I_3_ nanorods showed a weak yellow emission positioned at 553 nm with a PLQY of 5% and the 0D Cs_3_Cu_2_I_5_ nanocrystals exhibited a bright blue emission located at 441 nm with PLQY up to 67%. The satisfactory PLQY of Cs_3_Cu_2_I_5_ was ascribed to the 0D electronic dimension which confined excitons in spatially isolated polyhedra and resulted in a larger exciton binding energy. Above Cu(I)‐based halides with high PLQY show a great promise in the LED application. However, the peak wavelength of the excitation spectrum is located in the deep ultraviolet range (200–350 nm), which adds to the difficulty for LED applications. Cu(I)‐based halide materials with long excitation wavelengths are needed. Tang's group recently reported that 1D Rb_2_CuBr_3_ crystals with violet emission exhibited a great promise in the X‐ray scintillator detectors due to their near‐unity PLQY and large Stokes shift.^[^ ^111^ ^]^ These materials showed broad emission arising from self‐trapped excitons which were widely presented in low‐dimensional perovskites as depicted above.^[^ ^177^ ^]^ Recently, all‐inorganic Cu(I)‐based rare‐earth halide material, Rb_8_CuSc_3_Cl_18_, has been synthesized via a solid‐state reaction method by Yang's group. Herein, two Cu(I) ions are connected to the three rare‐earth halide octahedra to form a paddle‐wheel‐like cluster, which contributes to a strong blue PL emission as shown in Figure 10d,e, where Cu(I) adopts 3‐coordination environment.^[^ ^178^ ^]^

Apart from Cu‐based halide materials, there are some other metal‐based halide perovskites reported. Cs_2_TiBr_6_ vacancy‐ordered double perovskites thin films synthesized by a facile low‐temperature vapor‐based method showed bright luminescence centered at 704 nm under the excitation of 395 nm, which was attributed to its band‐edge emission.^[^ ^97^ ^]^ Concurrently, single crystals of Cs_2_PdBr_6_ vacancy‐ordered defect‐variant perovskite displayed emission at 772 nm.^[^ ^179^ ^]^ Lee's group reported rare‐earth‐element ytterbium substituted lead‐free halide perovskite nanocrystals of CsYbI_3_ synthesized by the hot injection method. The cubic perovskite CsYbI_3_ nanocrystals exhibited a strong PL at 671 nm as shown in Figure 10f and a high PLQY of 58% under the excitation of 450 nm.^[^ ^180^ ^]^ Yang's group synthesized CsEuCl_3_ nanocrystals with a blue emission at 435 nm and a narrow FWHM of 19 nm.^[^ ^181^ ^]^ Among lead‐free halide perovskites, CsEuCl_3_ possesses the narrowest FWHM, which shows some promise in display applications. The development of CsYbI_3_ and CsEuCl_3_ inspires new type lead‐free halide perovskite materials via rare‐earth substitutes. Su's group reported that red‐emitting Cs_2_InBr_5_·H_2_O single‐crystal demonstrated a broad emission at 695 nm with a high PLQY of 33% (Figure 10g).^[^ ^72^ ^]^ Interestingly, Cs_2_InBr_5_·H_2_O demonstrated a different emission color under additional water exposure, which shows the potential for water detection. The effect of octahedral ligands (H_2_O or Cl^−^) on STE emission with different emission wavelengths was investigated in Sb‐doped Rb_3_InCl_6_ and Sb‐doped Rb_3_InCl_5_(H_2_O) via controlling the distortion of the octahedron.^[^ ^182^ ^]^ Meantime, the study also suggests that the greater degree of distortion of the octahedron, the more red‐shift in STE emission, which provides an effective method to adjust the STE emission color.

Besides all‐inorganic In‐based perovskites reported, an organic–inorganic In‐based (C_4_H_14_N_2_)_2_In_2_Br_10_ single crystal was synthesized via a facile solution‐phase method by Kuang's group. The single crystal (C_4_H_14_N_2_)_2_In_2_Br_10_ demonstrated a broad orange‐red PL band from 500 nm to near‐infrared region with a long lifetime of 3.2 µs and a large Stokes shift over 300 nm.^[^ ^183^ ^]^

Mn‐based halide perovskites were also reported.^[^ ^184^ ^]^ (Pyrrolidinium)MnCl_3_ was demonstrated by Xiong's group, which demonstrated a highly efficient red‐light emission under UV excitation as shown in Figure 10h.^[^ ^185^ ^]^ Strikingly, (Pyrrolidinium)MnCl_3_ possesses ferroelectric properties, extending the applications to the field of ferroelectric luminescence or multifunctional devices.

Based on the above discussion, the optical properties and synthesis methods of a large proportion of currently reported lead‐free halide perovskite materials are summarized in **Table** **3**.

**Table 3:** Optical properties and synthesis methods of lead‐free halide perovskites (AVC, anti‐solvent vapor‐assisted crystallization; TLM, temperature lowering method; VHE, vapor halide exchange; VSA, vapor saturation of antisolvent;ASRP, antisolvent reprecipitation method LARP, ligand‐assisted reprecipitation method; SEM, slow evaporation method; NCs, nanocrystals; SCs, single crystals; BCs, bulk crystals; TF, thin films; p‐FPEA, 4‐fluorophenyl‐ethylamine; HMD, hexamethylene diamine; TTA, tetraethylammonium; TEBA: benzyltriethylammonium)

	Perovskite	Abs [nm]	PL [nm]	PLQY [%]	FWHM [nm]	Method	Refs.
3D	CsSnCl_3_ NCs	588	625	N/A	32	Solvothermal	^[^ ^119^ ^]^
3D	CsSnBr_3_ NCs	622	N/A	N/A	32	Solvothermal	^[^ ^119^ ^]^
3D	CsSnI_3_ NCs	668	709	N/A	32	Solvothermal	^[^ ^119^ ^]^
3D	CsSnCl_3_ NCs	420	490	≤0.14	N/A	Hot injection	^[^ ^33b^ ^]^
3D	CsSnBr_3_ NCs	610	660	≤0.14	N/A	Hot injection	^[^ ^33b^ ^]^
3D	CsSnI_3_ NCs	750	945	≤0.14	N/A	Hot injection	^[^ ^33b^ ^]^
3D	Cs_2_SnI_6_ NCs	N/A	620	≤0.48	49	Hot injection	^[^ ^66a^ ^]^
3D	Cs_2_SnCl_6_:Bi^3+^ BCs	N/A	455	78.9	66	Hydrothermal	^[^ ^16e^ ^]^
3D	Cs_2_SnCl_6_:Sb^3+^ BCs	N/A	602	37	101	Hydrothermal	^[^ ^187^ ^]^
2D	PEA_2_SnI_4_ NCs	N/A	640	6.40 ± 0.14	36	ASRP	^[^ ^128^ ^]^
2D	TEA_2_SnI_4_ NCs	624	645	18.85 ± 2.17	32.4	ASRP	^[^ ^44^ ^]^
2D	p‐FPEA_2_SnI_4_ NCs	621.2	640	9.94 ± 1.23	30.8	ASRP	^[^ ^44^ ^]^
2D	(OCTAm)_2_SnX_4_ BCs	350	600	95 ± 5	136	Solution process	^[^ ^56^ ^]^
2D	(OAm)_2_SnBr_4_ BCs	N/A	620	88	140	Hot injection	^[^ ^86^ ^]^
2D	(PEAI)_3.5_(CsI)_5_(SnI_2_)_4.5_ TFs	N/A	920	18	N/A	Spin‐coating	^[^ ^188^ ^]^
2D	(C_8_H_17_NH_3_)_2_SnBr_4_ BCs	N/A	596	98	N/A	Solution process	^[^ ^189^ ^]^
1D	(DAO)Sn_2_I_6_ SCs	N/A	634	20.3	142	Solution process	^[^ ^135^ ^]^
1D	(DAO)Sn_2_I_6_ TFs	N/A	–	36	145	Solution process	^[^ ^135^ ^]^
0D	(C_4_N_2_H_14_Br)_4_SnBr_6_ SCs	355	570	95 ± 5	105	Solution process	^[^ ^132^ ^]^
0D	(C_4_N_2_H_14_I_4_)SnI_6_ SCs	410	620	75 ± 4	118	Solution process	^[^ ^132^ ^]^
0D	Cs_4_SnBr_6_ powder	N/A	540	15 ± 5	N/A	Solid‐state process	^[^ ^133^ ^]^
0D	Cs_4_SnBr_6_ BCs	320	524	20	100	ASRP	^[^ ^190^ ^]^
0D	Cs_3_KSnBr_6_ BCs	320	500	35	100	ASRP	^[^ ^190^ ^]^
0D	Cs_4_SnBr_6_ NCs	N/A	530	0.8	45	Hot injection	^[^ ^191^ ^]^
0D	Bmpip_2_SnBr_4_ SCs	N/A	666	75	N/A	TLM	^[^ ^134^ ^]^
0D	Bmpip_2_SnI_4_ SCs	N/A	730	35	N/A	TLM	^[^ ^134^ ^]^
0D	HMD_3_SnBr_8_ BCs	N/A	601	86 ± 2	128	Antisolvent method	^[^ ^192^ ^]^
2D	(C_4_H_9_NH_3_)_2_GeI_4_ SCs	N/A	690	N/A	180	Solution process	^[^ ^137^ ^]^
2D	(PEA)_2_GeI_4_ BCs	N/A	613	N/A	98.4	Solution process	^[^ ^138^ ^]^
0D	Bmpip_2_GeBr_4_ SCs	N/A	670	≤1	N/A	Solution process	^[^ ^134^ ^]^
2D	MA_3_Bi_2_Br_9_ SCs	N/A	550	N/A	100	SEM	^[^ ^143^ ^]^
1D	MA_3_Bi_2_Cl_9_ NCs	N/A	360	15	50	Co‐LARP	^[^ ^143^ ^]^
2D	MA_3_Bi_2_Br_9_ NCs	376	423	12	62	Co‐LARP	^[^ ^143^ ^]^
2D	MA_3_Bi_2_I_9_ NCs	N/A	540	0.03	91	Co‐LARP	^[^ ^143^ ^]^
2D	MA_3_Bi_2_Br_9_‐Cl NCs	388	422	54.1	N/A	Co‐LARP	^[^ ^59^ ^]^
2D	Cs_3_Bi_2_Br_9_ NCs	N/A	460	4.5	45	Co‐LARP	^[^ ^144^ ^]^
2D	Cs_3_Bi_2_Cl_9_ NCs	N/A	393	26.4	59	Co‐LARP	^[^ ^58^ ^]^
2D	Cs_3_Bi_2_Br_9_ NCs	N/A	410	19.4	48	Co‐LARP	^[^ ^58^ ^]^
2D	Cs_3_Bi_2_I_9_ NCs	N/A	545	0.018	70	Co‐LARP	^[^ ^58^ ^]^
2D	FA_3_Bi_2_Br_9_ NCs	404	437	52	65	Co‐LARP	^[^ ^193^ ^]^
0D	Rb_7_Bi_3_Cl_16_ NCs	N/A	437	28.43	93	LARP	^[^ ^79^ ^]^
0D	(C_8_NH_12_)_4_BiBr_7_•H_2_O SCs	400	450	0.7	N/A	TLM	^[^ ^78^ ^]^
0D	(C8NH12)4Bi0.57Sb0.43Br7•H_2_O SCs	N/A	400–850	4.5	N/A	TLM	^[^ ^78^ ^]^
0D	(C_9_NH_20_)_2_SbCl_5_ SCs	N/A	590	98 ± 2	119	Solution process	^[^ ^132^ ^]^
0D	(Ph4P)_2_SbCl_5_ SCs	N/A	648	87 ± 2	136	AVC	^[^ ^82^ ^]^
0D	(TTA)_2_SbCl_5_ powder	N/A	625	86	140	ASRP	^[^ ^194^ ^]^
0D	(TEBA)_2_SbCl_5_ powder	N/A	590	98	135	ASRP	^[^ ^194^ ^]^
2D	Cs_3_Sb_2_Cl_9_ NCs	N/A	370	11	52	LARP	^[^ ^80^ ^]^
2D	Cs_3_Sb_2_Br_9_ NCs	N/A	410	46	41	LARP	^[^ ^80^ ^]^
2D	Cs_3_Sb_2_I_9_ NCs	N/A	560	23	56	LARP	^[^ ^80^ ^]^
2D	Cs_3_Sb_2_I_9_ TFs	N/A	750	N/A	120	VHE	^[^ ^156^ ^]^
2D	Cs_3_Sb_2_Br_9_ NCs	368	409	51.2	N/A	ASRP	^[^ ^66b^ ^]^
2D	(NH_4_)_3_Sb_2_I_9_ TFs	645	639	N/A	N/A	AVC	^[^ ^155^ ^]^
3D	Cs_2_AgBiCl_6_ NCs	N/A	395	6.7	68	ASRP	^[^ ^62^ ^]^
3D	Cs_2_AgBiBr_6_ NCs	N/A	465	0.7	82	ASRP	^[^ ^62^ ^]^
3D	Cs_2_AgBiI_6_ NCs	N/A	575	<0.1	69	ASRP	^[^ ^62^ ^]^
3D	Cs_2_AgBiCl_6_: Mn^2+^ NCs	N/A	600	<1	N/A	Hot injection	^[^ ^196^ ^]^
3D	Cs_2_Ag_0.17_Na_0.83_In_0.88_Bi_0.12_Cl_6_ NCs	N/A	557	64	153	ASRP	^[^ ^168^ ^]^
3D	Cs_2_AgIn_0.9_Bi_0.1_Cl_6_ NCs	372	395/570	36.6	N/A	ASRP	^[^ ^24^ ^]^
3D	Cs_2_NaInCl_6_: Ag^+^ NCs	269	535	31.1	N/A	Hot injection	^[^ ^85^ ^]^
3D	Cs_2_AgInCl_6_ NCs	350	560	≈1.6 ± 1	N/A	Hot injection	^[^ ^66c^ ^]^
3D	Cs_2_AgInCl_6_:Mn^2+^ NCs	350	620	≈16 ± 4	N/A	Hot injection	^[^ ^66c^ ^]^
3D	Cs_2_AgInCl_6_:Bi^3+^ NCs	368	580	11.4	N/A	Hot injection	^[^ ^84b^ ^]^
3D	Cs_2_AgInCl_6_:Yb^3+^/Er^3+^ NCs	N/A	996/1537	N/A	N/A	Hot injection	^[^ ^167^ ^]^
3D	Cs_2_Ag_1−_ *_x_*Na*_x_*InCl_6_:Bi^3+^ NCs	N/A	N/A	22	N/A	Hot injection	^[^ ^197^ ^]^
3D	Cs_2_Ag_0.6_Na_0.4_InCl_6_: 0.04Bi^3+^ BCs	N/A	565	86 ± 5	N/A	Hydrothermal	^[^ ^60^ ^]^
3D	Cs_2_AgInCl_6_: 0.9% Mn^2+^ BCs	N/A	630	3–5	N/A	Solution process	^[^ ^163^ ^]^
–	Cs_2_CuBr_4_ NCs	360	393	37.5	74	LARP	^[^ ^175^ ^]^
‐	Cs_2_CuCl_4_ NCs	N/A	388	51.82	68	LARP	^[^ ^175^ ^]^
–	CsCuBr_2_ MCs	N/A	495	N/A	70	Solution process	^[^ ^198^ ^]^
1D	CsCu_2_I_3_ NRs	330	553	5	N/A	Hot injection	^[^ ^177^ ^]^
0D	Cs_3_Cu_2_I_5_ NCs	285	441	67	N/A	Hot injection	^[^ ^177^ ^]^
0D	Cs_3_Cu_2_Br_5_ NCs	277	454	18.3	82	Hot injection	^[^ ^177^ ^]^
1D	CsCu_2_Cl_3_ powder	N/A	527	48	102	Solid‐state reaction	^[^ ^200^ ^]^
1D	CsCu_2_Br_3_ powder	N/A	533	18.3	106	Solid‐state reaction	^[^ ^200^ ^]^
1D	CsCu_2_I_3_ powder	N/A	576	3.23	126	Solid‐state reaction	^[^ ^200^ ^]^
0D	Cs_3_Cu_2_Br_5_ powder	N/A	455	50.1	75	Solid‐state reaction	^[^ ^176^ ^]^
0D	Cs_3_Cu_2_I_5_ powder	N/A	443	98.7	99	Solid‐state reaction	^[^ ^176^ ^]^
0D	Cs_3_Cu_2_I_5_ SCs	N/A	445	91.2	N/A	VSA	^[^ ^61^ ^]^
0D	Cs_3_Cu_2_I_5_ TFs	N/A	445	62.1	N/A	Spin‐coating	^[^ ^61^ ^]^
0D	Cs_3_Cu_2_I_5_ NCs	285	445	29.2	N/A	Hot injection	^[^ ^201^ ^]^
0D	Cs_3_Cu_2_Br_5_ NCs	269	461	16.9	N/A	Hot injection	^[^ ^201^ ^]^
0D	Cs_3_Cu_2_Cl_5_ NCs	259	527	48.7	N/A	Hot injection	^[^ ^201^ ^]^
1D	CsCu_2_I_3_ TFs	N/A	550	20.6	100	Antisolvent method	^[^ ^211^ ^]^
1D	CsCu_2_I_3_ SCs	N/A	568	15.7	75	Antisolvent method	^[^ ^202^ ^]^
0D	Cs_2_InBr_5_ ·H_2_O SCs	N/A	695	33	N/A	TLM	^[^ ^72^ ^]^
0D	Rb_2_InCl_5_(H_2_O): Sb^3+^ SCs	324	600	90	N/A	Hydrothermal	^[^ ^182^ ^]^
0D	Rb_2_InCl_6_: Sb^3+^ SCs	N/A	497	95	N/A	Solvent thermal	^[^ ^182^ ^]^
0D	(C_4_H_14_N_2_)_2_In_2_Br_10_ SCs	335	670	3	N/A	Solution process	^[^ ^183^ ^]^
3D	CsYbI_3_ NCs	N/A	671	58	47	Hot injection	^[^ ^180^ ^]^
3D	Cs_2_TiBr_6_ TFs	N/A	704	N/A	N/A	Vapor deposition	^[^ ^97^ ^]^
3D	Cs_2_PdBr_6_ SCs	784	772	N/A	N/A	Solution process	^[^ ^179^ ^]^
1D	(Pyrrolidinium)MnCl_3_ SCs	N/A	637	56	N/A	SEM	^[^ ^185^ ^]^

So far, there are many lead‐free halide perovskites reported. It is crucial to understand the structure–property relationship and the underlying mechanisms in terms of reported lead‐free halide perovskites. That will provide some effective methods to improve performance. Besides, combining theoretical and experimental approaches to develop new‐type lead‐free halide perovskites is also very effective.

## 5 LED Applications

In addition to the considerations of materials, the device designs are also crucial to the development of LED applications. There are two mechanisms for LED light generation: PL and electroluminescence (EL).^[^ ^203^ ^]^ Correspondingly, lead‐free halide perovskite materials are applied to phosphor‐converted LEDs and electroluminescent LEDs, as summarized in **Tables** **4** and **5**, respectively.

**Table 4:** Phosphor‐converted LEDs from lead‐free halide perovskites

Emitting material	Device structure	CCT [K]	CIE coordinates	CRI	Stability	Refs.
Cs_2_SnCl_6_:2.75%Bi^3+^	Cs_2_SnCl_6_:2.75%Bi^3+^/ Ba_2_Sr_2_SiO_4_:Eu^2+^/ GaAlSiN_3_:Eu^2+^/365 nm chip (silicone)	4486	(0.36, 0.37)	N/A	N/A	^[^ ^16e^ ^]^
Cs_2_Ag_0.6_Na_0.4_InCl_6_:0.04Bi^3+^	Cs_2_Ag_0.6_Na_0.4_InCl_6_:0.04Bi^3+^/ultraviolet chip	4054	(0.396, 0.448)	N/A	≈100% (1000 h working on LED)	^[^ ^60^ ^]^
(OCTAm)_2_SnBr_4_	(OCTAm)SnBr_4_/BaMgAl_10_O_17_:Eu^2+^/G2762/365 nm chip	5635 6530	(0.33, 0.26) (0.33, 0.31)	81 89	N/A	^[^ ^56^ ^]^
HMD_3_SnBr_8_	HMD_3_SnBr_8_/BaMgAl_10_O_17_:Eu^2+^, Mn^2+^/ UV chip	5700	N/A	94	≈100% (24 h working on LED)	^[^ ^192^ ^]^
Cs_3_Bi_2_Br_9_	Cs_3_Bi_2_Br_9_ NCs with silica composites/Y_3_Al_5_O_12_/GaN chip	8477.1	(0.29,0.30)	N/A	78% (UV 16 h) 68% (60 °C 15 h)	^[^ ^58^ ^]^
Cs_2_SnCl_6_ :0.59%Sb^3+^	Cs_2_SnCl_6_:0.59%Sb^3+^/ Cs_2_SnCl_6_:2.75%Bi^3+^/ Ba_2_Sr_2_SiO_4_:Eu^2+^/380 nm chip(TEOS)	6815	(0.30, 0.37)	81	N/A	^[^ ^187^ ^]^
(C_4_N_2_H_14_Br)_4_SnBr_6_	(C_4_N_2_H_14_Br)_4_SnBr_6_/ BaMgAl_10_O_17_:Eu^2+^(PDMS)/340 nm chip	4946	(0.35, 0.39)	70	≈100% (6 h working on LED)	^[^ ^132^ ^]^

**Table 5:** Electroluminescent LEDs from lead‐free halide perovskites

Emitting material (EM)	Device structure	EL [nm]	EQE_max_ [%]	CE_max_ [cd A^−1^]	*L*_max_ [cd m^−2^]	Refs.
CsSnI_3_	ITO/PEDOT/EM/PBD/LiF/Al	950	3.8	N/A	N/A	^[^ ^229^ ^]^
MASn(Br_0.5_I_0.5_)_3_	ITO/PEDOT:PSS/EM/F8/Ca/Ag	667	0.007	N/A	N/A	^[^ ^206^ ^]^
MASn(Br_0.4_I_0.6_)_3_	ITO/PEDOT:PSS/EM/F8/Ca/Ag	780	0.019	N/A	N/A	^[^ ^206^ ^]^
MASn(Br_0.3_I_0.7_)_3_	ITO/PEDOT:PSS/EM/F8/Ca/Ag	825	0.046	N/A	N/A	^[^ ^206^ ^]^
MASn(Br_0.2_I_0.8_)_3_	ITO/PEDOT:PSS/EM/F8/Ca/Ag	868	0.058	N/A	N/A	^[^ ^206^ ^]^
MASn(Br_0.1_I_0.9_)_3_	ITO/PEDOT:PSS/EM/F8/Ca/Ag	896	0.11	N/A	N/A	^[^ ^206^ ^]^
MASnI_3_	ITO/PEDOT:PSS/EM/F8/Ca/Ag	945	0.72	N/A	N/A	^[^ ^206^ ^]^
PEA_2_SnI_4_	ITO/PEDOT:PSS/EM/F8/LiF/Al	618	N/A	0.029	0.15	^[^ ^129^ ^]^
PEA_2_SnI_4_	(ITO)/PEDOT:PSS /EM/TPBi/LiF/Al	633	0.3	N/A	70	^[^ ^136^
TEA_2_SnI_4_	ITO/TPBi/LiF/EM/TPBi/LiF/Al	638	0.62	N/A	322	^[^ ^207^ ^]^
(OAm)_2_SnBr_4_	ITO/PEI‐ZnO/ EM/TCTA/MoO_3_/Au	625	0.1	0.029	350	^[^ ^86^ ^]^
(PEAI)_3.5_(CsI)_5_(SnI_2_)_4.5_	ITO/PVK/EM/TmPyPB/LiF/Al	920	3	N/A	N/A	^[^ ^188^ ^]^
Cs_2_Ag_0.6_Na_0.4_InCl_6_:Bi^3+^	PEIE‐ITO/PEIE‐ZnO/EM/TAPC/MoO_3_/Al	N/A	N/A	0.11	N/A	^[^ ^60^ ^]^
CsCuBr_2_	Ag/EM/p‐Si/In‐Ga	527	N/A	N/A	N/A	^[^ ^198^ ^]^
Cs_3_Cu_2_I_5_	ITO/ZSO/EM/NPD/MoOx/Ag	440	N/A	N/A	10	^[^ ^61^ ^]^
CsCu_2_I_3_	ITO/PEDOT:PSS/Poly‐TPD/EM /TPBi/LiF/Al	550	0.17	0.46	47.5	^[^ ^228^ ^]^
Cs_3_Cu_2_I_5_	Al/LiF/TPBi/EM/NiO/ITO	445	1.12	N/A	263.2	^[^ ^208^ ^]^
Cs_3_Cu_2_I_5_	ITO/PEDOT:PSS:PFI/ Cs_3_Cu_2_I_5_‐poly‐HEMA film/TPBI/LiF /Al	N/A	0.27	N/A	140	^[^ ^209^ ^]^
Cs_3_Sb_2_I_9_	Glass/ITO/PEDOT:PSS/EM/TPBi/LiF/Al	N/A	10^−8^	N/A	N/A	^[^ ^156^ ^]^
Cs_3_Sb_2_Br_9_	ITO/ZnO:PEI/EM/TCTA/MoO_3_/Al	408	∼0.206	N/A	N/A	^[^ ^66b^ ^]^
CsSnBr_3_	Glass/ITO/LiF/EM/LiF/ZnS/Ag	672	0.34	N/A	N/A	^[^ ^101^ ^]^

### 5.1 Phosphor‐Converted LEDs

The phosphor‐converted LEDs (pc‐LEDs) remain to be a leading position because of their superior luminous efficiency, long operation time, and simple fabrication. The choice of phosphors has a vital influence on the performance of pc‐LEDs. As we all know, the rare‐earth‐based phosphors are the majority in the market, and have made significant advances in pc‐LEDs. However, phosphors based on rare‐earths also face some problems, such as high cost and recycle difficulty. Low‐cost halide perovskites have demonstrated a great promise in pc‐LEDs due to their extremely excellent optical properties, including high PLQY, tunable emission, and high color purity. There is a large number of halide perovskites applied to light emission and displays.^[^ ^204^ ^]^ In order to solve the toxicity issue of lead, developing environmentally benign lead‐free perovskite phosphors is necessary.

**Figure 11:**
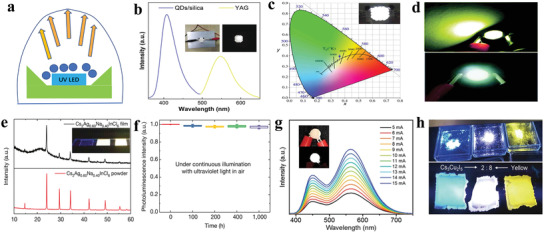
a) Schematic of phosphor‐converted LED. b) EL spectrum of the WLED by integrating blue‐emitting Cs_3_Bi_2_Br_9_ NCs and yellow‐emitting rare‐earth phosphor Y_3_Al_5_O_12_. Inset: device photos of NCs/silica composites combined with Y_3_Al_5_O_12_ (off and on). Reproduced with permission.^[^ ^58^ ^]^ Copyright 2017, Wiley‐VCH. c) CIE color coordinates of WLED device combined by Cs_2_SnCl_6_:2.75%Bi^3+^/Ba_2_Sr_2_SiO_4_:Eu^2+^/GaAlSiN_3_:Eu^2+^/365 chip and inset image of operating LED. Reproduced with permission.^[^ ^16e^ ^]^ Copyright 2018, Wiley‐VCH. d) 365 nm UV pumped single yellow‐emitting (OCTAm)_2_SnBr_4_ LED and WLED fabricated by (OCTAm)SnBr_4_/BaMgAl_10_O_17_:Eu^2+^/G2762/365 nm UV chip. Reproduced with permission.^[^ ^56^ ^]^ Copyright 2019, Royal Society of Chemistry. e) XRD patterns of a Cs_2_Ag_0.6_Na_0.4_InCl_6_ films (top) and powder (bottom). The inset displays a 300 nm thick quartz substrate and 500 nm thick Cs_2_Ag_0.6_Na_0.4_InCl_6_ films under the excitation of 254 nm. f) Operational stability of Cs_2_Ag_0.6_Na_0.4_InCl_6_ devices, measured in air without any encapsulation. Reproduced with permission.^[^ ^60^ ^]^ Copyright 2018, Springer Nature. g) EL spectra of a WLED at different driving currents, the insets show the device when off and on. Reproduced with permission.^[^ ^132^ ^]^ Copyright 2018, Royal Society of Chemistry. h) Photograph of Cs_3_Cu_2_I_5_, a yellow phosphor, and its mixtures (from left to right: Cs_3_Cu_2_I_5_, mixture of 2:8, yellow phosphor) under UV excitation (top) and films prepared by kneading the powders into polydimethylsiloxane matrix (bottom). Reproduced with permission.^[^ ^61^ ^]^ Copyright 2018, Wiley‐VCH.

As shown in **Figure** **11**a, phosphor‐converted LEDs are encapsulated by integrating the LED chips and emissive materials, which are widely applied to the fields of lighting and backlight down‐converters for liquid crystal displays (LCDs) owing to their superior characteristics.^[^ ^64^, ^205^ ^]^ Herein, high energy light sources coming from LED chips (such as InGaN, GaN) are exploited to excite the emissive materials, which can emit lights corresponding to the materials’ optical bandgaps. Obviously, the encapsulants and encapsulation processes affect the performance, duration and stability of the devices.^[^ ^64^ ^]^

Several parameters are considered to be significant for depicting the performance of pc‐LEDs, including luminous efficacy (LE), color rendering index (CRI), correlated color temperature (CCT) and CIE color coordinates. LE is a measure of how well a light source produces visible light and equals the ratio of luminous flux (lumens, lm) to power (W), which is the foremost specification for pc‐LEDs. Generally, LE is used to depict the power consumption of a light source, which is dependent on two factors: the efficiency from electrical power to optical power and the conversion factor from optical power to luminous flux. CRI stands for the ability of a light source to reveal the colors of various objects truly in comparison with an ideal or natural light source. Light sources with a high CRI are desirable in displays and lighting. CCT is the temperature of an ideal black‐body radiates light of a color comparable to that of the light source. Generally, the value of CCT over 5000 K is considered as cold white light, and lower CCT is considered as cool white light. CIE color coordinates are quantitative links between wavelengths and human eye perceived colors, usually with the CIE 1931 chromaticity diagram. Besides, CIE 1976 chromaticity diagram was established to solve the uniformity problem of the 1931 chromaticity diagram.

Tang's group fabricated white light‐emitting devices (WLEDs) via a combination of Cs_3_Bi_2_Br_9_ nanocrystal/silica composites and yellow‐emitting phosphors under a GaN chip excitation (Figure 11b). The WLEDs demonstrated CIE color coordinates of (0.29, 0.30) and the CCT of 8477.1 K.^[^ ^58^ ^]^ As shown in Figure 11c, blue‐emitting Cs_2_SnCl_6_:2.75%Bi^3+^, yellow‐emitting phosphors (Ba_2_Sr_2_SiO_4_:Eu^2+^ and GaAlSiN_3_:Eu^2+^) and a 365 nm chip were combined to construct WLEDs, which displayed CIE of (0.36, 0.37) and a warm white with a CCT of 4486 K.^[^ ^16e^ ^]^ The WLEDs with a high color rending index (CRI = 81) were reported by Li et al., which consisted of orange‐emitting Cs_2_SnCl_6_:0.59%Sb^3+^, blue‐emitting Cs_2_SnCl_6_:2.75%Bi^3+^ and green‐emitting Ba_2_Sr_2_SiO_4_:Eu^2+^ at a ratio of 1:1:1 encapsulated by a silicone polymer with a commercial 380 nm chip.^[^ ^187^ ^]^

Deng's group synthesized yellow‐emitting (OCTAm)_2_SnBr_4_ for WLEDs, which prepared the “warm” or “cool” WLEDs via adjusting the material ratio.^[^ ^56^ ^]^ As shown in Figure 11d, single yellow phosphor (OCTAm)_2_SnBr_4_ pumped by UV (365 nm) and white LED devices fabricated by phosphors mixtures were demonstrated. It is noteworthy that a single white emitter Cs_2_Ag_0.6_Na_0.4_InCl_6_:0.04Bi^3+^ was fabricated into WLEDs by directly pressing the materials on a commercial ultraviolet LED chip without any encapsulation for protection (Figure 11e). Excitingly, the WLEDs presented negligible decomposition when they were operated at about 5000 cd m^−2^ for over 1000 h in air, as shown in Figure 11f.^[^ ^60^ ^]^ A mixture of 0D (C_4_N_2_H_14_Br)_4_SnBr_6_ and blue phosphor (BaMgAl_10_O_17_:Eu^2+^) at a 1:1 ratio showed decent white light with a CCT of 4946 K, a CRI of 70 and the CIE coordinates of (0.35, 0.39). As can be seen from Figure 11g, the excellent color stability was presented with different operation currents, which might be attributed to no energy transfer between the two phosphors.^[^ ^132^ ^]^ Blue‐emitting Cs_3_Cu_2_I_5_ with a yellow‐emitting phosphor were mixed into a polydimethylsiloxane matrix, which demonstrated bright white emission under UV illumination (Figure 11h).^[^ ^61^ ^]^

### 5.2 Electroluminescent LEDs

**Figure 12:**
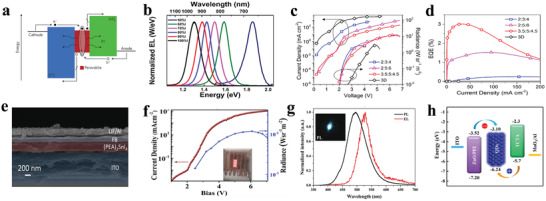
a) Schematic representation of typical electroluminescent device structure. Reproduced with permission.^[^ ^221^ ^]^ Copyright 2016, Springer Nature. b) Normalized EL spectra of ITO/PEDOT:PSS/CH_3_NH_3_Sn(Br_1‐_ *_x_*I*_x_*)_3_/F8/Ca/Ag LED. Reproduced with permission.^[^ ^206^ ^]^ Copyright 2016, American Chemical Society. c) Dependence of the current density and radiance on the driving voltage for (PEAI)*_x_*(CsI)*_y_*(SnI_2_)*_z_*. d) EQE values versus current density for (PEAI)*_x_*(CsI)*_y_*(SnI_2_)*_z_*. For the (PEAI)_3.5_(CsI)_5_(SnI_2_)_4.5_ MQW‐based LED, a maximum EQE of 3% is achieved. Reproduced with permission.^[^ ^188^ ^]^ Copyright 2017, American Chemical Society. e) Cross‐sectional picture of the ITO/PEDOT:PSS/(PEA)_2_SnI_4_/F8/LiF/Al device. Reproduced with permission.^[^ ^129^ ^]^ Copyright 2017, American Chemical Society. f) Current density versus voltage (*J*−*V*) and radiance versus voltage characteristics for glass/ITO/PEDOT:PSS/Cs_3_Sb_2_I_9_ thin film/TPBi/LiF/Al. Reproduced with permission.^[^ ^156^ ^]^ Copyright 2019, American Chemical Society. g) PL spectrum of the CsCuBr_2_ micro‐crosses on the Si substrate and EL spectrum of the LED at 2.8 V. Inset: a PL photograph of the CsCuBr_2_ micro‐crosses. Reproduced with permission.^[^ ^198^ ^]^ Copyright 2019, Royal Society of Chemistry. h) Simplified energy levels of each layer of the heterostructure in LED based on Cs_3_Sb_2_Br_9_ nanocrystals. Reproduced with permission.^[^ ^195^ ^]^ Copyright 2019, American Chemical Society.

Electroluminescent devices can emit light when the injected electrons and holes encounter and radiatively recombine in the active layer. In fact, solution‐processed lead‐based halide perovskites have been widely investigated in electroluminescent LEDs with a considerable progress.^[^ ^210^ ^]^ The EQE of lead‐based halide perovskite LEDs have been rapidly improved from the initial 0.76% to over 20% in recent years. So far, the maximum EQE of perovskite LEDs for green, red and infrared emission have reached 28.2%, 21.3%, 21.6%, respectively.^[^ ^12^, ^211^ ^]^ As for the blue‐emitting LEDs, the maximum EQE has also reached 9.5%.^[^ ^212^ ^]^ A typical electroluminescent device structure is demonstrated in **Figure** **12**a. The structure consists of an anode, a hole transport layer (HTL), an emitter layer, an electron transport layer (ETL) and a cathode. Under an applied voltage, the electrons and holes can be injected from cathode and anode through ETL and HTL to emitter layer to form excitons and subsequently emit light.^[^ ^213^ ^]^ There are two significant specifications related to LED performance: PLQY and EL intensity. The PLQY of the emitter layer is widely used to describe the optoelectronic quality of the emitter layer.^[^ ^214^ ^]^ However, consideration for PLQY of the active layer alone is not sufficient to achieve good performance. The internal quantum efficiency (IQE) is defined as the proportion between the number of photons emitted in the emitter layers and the number of electrons injected into the LED. To obtain a larger IQE, the nonradiative electron–hole recombination should be minimized and charges should not pass through the device in the absence of radiative recombination. Hence, the electron and hole blocking layers should be applied. The transport layers also serve as blocking layers to keep electrons and holes from escaping from the emissive layer.^[^ ^215^ ^]^ Moreover, there is a relationship EQE = *η*_0_ ∙ IQE, where *η*_0_ is an optical outcoupling coefficient, describing as how many emitted photons can be extracted from the LED into the free space or the fraction of emitted photons that are coupled out of the device. The higher EQE values of electroluminescent LEDs are actively pursued.

In addition, the figures of merit that evaluate the performance of LEDs also include current efficiency (CE), maximum luminance (*L*_max_), turn‐on voltage (*V*_on_), and operational stability. CE = *L*/*J*, where *L* and *J* are the luminance of the LED and the current density, respectively, and *V*_on_ is defined as the required voltage to make the luminescence of 1 cd m^−2^. These parameters are crucial to evaluate the performance of the electroluminescent LEDs.^[^ ^216^ ^]^

The carrier mobility must be emphasized in electroluminescent LEDs. The mobility is the speed that a charge carrier moves through a conductor in the presence of an electric field. The mobility of carriers and exciton binding energy are two important parameters for optoelectronic devices, which can influence the carrier recombination process. The efficient carrier injection and radiative recombination are in demand, whereas the nonradiative recombination originating from defects and Auger recombination should be avoided. The larger exciton binding energy and moderate mobility are beneficial for improving the radiative recombination. Low mobility can lead to electron aggregation at one side of the devices and hole aggregation at the other side, which will aggravate the Auger recombination. There is an exceptional review relevant to engineering charge transport in heterostructured materials.^[^ ^2f^ ^]^

So far, there are only a few reports on lead‐free halide perovskites for electroluminescent LED applications. Among them, infrared electroluminescent LEDs account for the majority. Early in 2016, Tan's group reported a series of electroluminescent LEDs based on CH_3_NH_3_Sn(Br_1‐_ *_x_*I*_x_*)_3_ (*x* = 0.5–1) thin films with the device structure of ITO/PEDOT:PSS/CH_3_NH_3_Sn(Br_1‐_ *_x_*I*_x_*)_3_/F8/Ca/Ag. As shown in Figure 12b, the EL peak ranged from 667 to 945 nm under different Br/I ratios, and a maximum EQE of 0.72% was obtained, when the emissive material was CH_3_NH_3_SnI_3_ thin films.^[^ ^206^ ^]^ As is known to all, high‐quality thin films are favorable for the LED performance. Chao's team reported that the infrared LEDs fabricated by high‐quality CsSnI_3_ thin films exhibited outstanding properties. The device structure was ITO/PEDOT:PSS/CsSnI_3_ thin films/F8/LiF/Al. The EL peak located at 950 nm with a maximum radiance of 40 W sr^−1^ m^−2^ and a maximum EQE of 3.8% was obtained.^[^ ^229^ ^]^

Quasi‐2D perovskites with multiple quantum‐well structures showed good stability, high PLQY and excellent performance in LEDs. The high PLQY can be ascribed to cascade energy transfer and exciton confinement.^[^ ^217^ ^]^ Expectantly, Sn‐based 2D perovskites were exploited to fabricate electroluminescent LEDs for the first time and exhibited good results. The synthesized (PEAI)*_x_*(CsI)*_y_*(SnI_2_)*_z_* uniform and stable thin films showed a series of EL spectra by adjusting the precursor ratio. The highest EQE of up to 3% at 920 nm with a radiance of 40 W sr^−1^ m^−2^ was achieved by (PEAI)_3.5_(CsI)_5_(SnI_2_)_4.5_ as shown in Figure 12c,d.

In addition to infrared electroluminescent LEDs, visible electroluminescent LEDs based on 2D Sn‐based perovskites were also studied by Zhang's and Haque's groups.^[^ ^86^, ^129^ ^]^ A bright orange‐emitting electroluminescent LED using (OAm)_2_SnBr_4_ with a device structure of ITO/PEI‐ZnO/perovskite/TCTA/MoO_3_/Au was fabricated. A maximum luminance of 350 cd m^−2^ was obtained, which is the highest brightness in lead‐free electroluminescent LEDs. A red LED with structure of ITO/PEDOT:PSS/(PEA)_2_SnI_4_/F8/LiF/Al (PEDOT:PSS and F8 were hole and electron injection layers, respectively) (Figure 12e) showed an EL peak at 618 nm with a current efficacy (CE) of 0.003 cd A^−1^ and luminance up to 0.15 cd m^−2^.^[^ ^129^ ^]^ The pure red‐emitting Sn‐based electroluminescent LEDs were reported and displayed the improved performance.^[^ ^143^ ^]^ When TEA_2_SnI_4_ served as the emission layer in the ITO/PEDOT:PSS/emission layer/TPBi/LiF/Al device, the electroluminescent LEDs demonstrated a low turn‐on voltage of 2.3 V, a maximum EQE of 0.62% and maximum luminance of 322 cd m^−2^.^[^ ^207^ ^]^ For the 2D perovskites applied to optoelectronic devices, the charge transport between the inorganic layers is limited due to the block of organic layers. To overcome this issue, a vertical crystal arrangement was adopted in the optoelectronic devices, which means that the inorganic layers are perpendicular to the substrate and facilitate the efficient charge carrier transport.^[^ ^126^ ^]^

Sb‐based perovskite thin films were first introduced in the electroluminescent LEDs by Chu's group, and the device structure was ITO/PEDOT:PSS/Cs_3_Sb_2_I_9_ thin film/TPBi/LiF/Al. Figure 12f demonstrates the rapid increase of current density under the condition of 2 V voltage, and the radiance of 0.012 W sr^−1^m^−2^ at 6 V.^[^ ^156^ ^]^ Yuan et al. fabricated a series of CsSnX_3_ thin films and utilized them to fabricate a red‐emitting electroluminescent LED, which had a current density as high as 915 A cm^−2^.^[^ ^101^ ^]^ In addition to the red‐emitting electroluminescent LEDs, green‐ and blue‐emitting electroluminescent LEDs were also reported using lead‐free perovskites. A green EL at 527 nm was observed via integrating CsCuBr_2_ micro‐crosses (MCs) in the device structure of Ag/CsCuBr_2_/p‐Si/In–Ga, as shown in Figure 12g. The peak shift between PL and EL was attributed to the different emission mechanisms.^[^ ^198^ ^]^ The PL originates from CsCuBr_2_ surface, whereas the EL is generated at the CsCuBr_2_ MC/p‐Si interface due to the recombination of electrons and holes. Hosono's group showed a blue electroluminescent LED based on Cs_3_Cu_2_I_5_ thin films, and the maximum luminance was approximately 10 cd m^−2^.^[^ ^61^ ^]^ Tang's group also reported an electroluminescent LED based on Cs_2_Ag_0.6_Na_0.4_InCl_6_:Bi^3+^ and demonstrated a low efficiency. Above‐mentioned electroluminescent LEDs based on Cs_3_Cu_2_I_5_ and Cs_2_Ag_0.6_Na_0.4_InCl_6_:Bi^3+^ were originated from STE emission. The low EQEs are due to the low carrier mobility owing to low electronic dimensionality, strong coupling to the lattice, and the poor charge injection because of the material's large bandgap.^[^ ^109a^ ^]^

Electroluminescent LEDs using lead‐free halide perovskite nanocrystals were first reported by Shi's group. As shown in Figure 12h, the suitable energy level structures were exploited, where TCTA served as the hole‐transporting layer and electron blocking layer owing to the suitable VBM and electron affinity. Electroluminescent LEDs using Cs_3_Sb_2_Br_9_ quantum dots manifested a violet emission at 408 nm and with an EQE of ≈0.206%.^[^ ^66b^ ^]^ There are reasons that these lead‐free perovskite nanocrystal electroluminescent LEDs are rare. On the one hand, it is due to the inferior characteristics of lead‐free perovskite nanocrystals including low PLQY, wide FWHM, and poor stability.^[^ ^218^ ^]^ On the other hand, the key obstacles of performance for nanocrystal film‐based devices were the presence of organic ligands and nonuniform films. The surface organic ligands serving as an insulating layer block charge injection into the emitter in LEDs. Besides, films of nanocrystals typically consist of agglomerates and clusters, which technically can be improved by using a nanocrystal solution with low concentration. Moreover, common surface ligand oleic acid and oleylamine, weakly bonded to nanocrystals, would degrade under device operating conditions.^[^ ^219^ ^]^ Hence, choosing proper ligands to improve the device's performance is significant, which has been reported in lead‐based halide perovskite devices.^[^ ^220^ ^]^ In addition, the nanocrystal film quality can be improved via interface engineering, such as a simple posttreatment utilizing PEI (polyethylenimine), which has been proven to be effective in obtaining high‐quality perovskite nanocrystal thin‐films.^[^ ^210^

## 6 Summary and Outlook

The lead‐free halide perovskite materials have drawn increasing attention due to the environmentally benign property in optoelectronic applications. In recent years, enormous efforts have been devoted to exploiting lead‐free halide perovskites. A large number of low‐toxic or nontoxic metal cations such as Sn (II), Sn (IV), Ge (II), Bi (III), Sb (III), Ag (I), Cu (II), and Cu (I), have replaced Pb (II). In this review, we highlighted the excellent characteristics of halide perovskites that benefit light generation, including the defect‐tolerance, bandgap type, and exciton binding energy. We summarized the synthesis methods of lead‐free halide perovskites, and systemically reviewed the optoelectronic properties of reported lead‐free halide perovskites, specially at different molecular dimensionalities with various forms of single crystals, thin films and nanocrystals. The LED applications of lead‐free halide perovskite were also delved.

However, there are still many issues to be solved in future research. Searching for lead‐free halide perovskites with high performance is still urgent and necessary. For the most reported lead‐free halide perovskites, the FWHM of emission is wider than lead halide perovskites, which is not suitable for display applications. More efforts should be made to develop lead‐free halide perovskites with narrow‐band emission for ultrahigh definition displays. The emissions of lead‐free halide perovskites originated from STEs have an excellent promise in WLED applications.^[^ ^60^ ^]^ In addition, we believe that lead‐free Sn‐based halide perovskites also demonstrate vast potential in near‐infrared emissions, which can be widely applied to plant growth, bioimaging, optical data communication, and night‐vision devices.

The performance of LEDs using lead‐free halide perovskite materials is far from those with lead halide perovskites. More efforts are needed to make significant advances in material preparations and device engineering on film/interlayer optimizations. The theoretical and computational material screening has been widely applied in the solar cells, which will give us some guidance to develop new lead‐free halide perovskites for other optoelectronic applications.^[^ ^222^ ^]^ As we all know, the first‐principles high‐throughput computational material screening is a useful tool to accelerate material discovery. Yang's group identified 23 organic–inorganic hybrid halide semiconductor candidates for light‐emitting materials through high‐throughput calculations.^[^ ^140^ ^]^ Recently. Zhang's group summarized the different screening criteria for the materials applied to various optoelectronic applications, including photovoltaic solar cells, photoelectrochemical cells, and LEDs.^[^ ^223^ ^]^ These selected materials can be synthesized via the proper methods. The investigations combined with theoretical, computational, and experimental methods will tremendously promote the development of lead‐free perovskite materials.^[^ ^224^ ^]^

For the phosphor‐converted LEDs, warm WLED lighting devices with long lifetimes are in demand. Hence, it is significant to develop the lead‐free halide perovskites with suitable excitation wavelengths and proper device encapsulation. For the electroluminescent LEDs, the lead‐free halide perovskites or lead‐free halide materials with high PLQY do not guarantee good LED performance. Exploiting lead‐free halide perovskites with high PLQY and excellent carrier mobilities is a final goal. Besides, the quality of lead‐free halide perovskite thin films cannot be ignored. Doping and post‐treatment are two significant strategies to improve perovskite thin film quality, which have been proven to be extremely effective in the field of perovskite solar cells.^[^ ^2^, ^225^ ^]^ The reported emissive layers consist mainly of low‐dimensional perovskites or low‐dimensional Cu‐based halide materials, including 0D Cs_3_Cu_2_I_5_, 1D CsCu_2_I_3_, 2D Cs_3_Sb_2_Br_9_, 2D (OAm)_2_SnBr_4_, 2D PEA_2_SnI_4_, 2D TEA_2_SnI_4_, 2D (PEAI)_3.5_(CsI)_5_(SnI_2_)_4.5_.^[^ ^66^, ^86^, ^136^, ^195^, ^206^, ^208^, ^228^ ^]^ Low‐dimensional perovskites or halide materials generally possess low electronic dimensionalities and result in low carrier mobilities and undesirable performances. Besides, the large organic group in low‐dimensional perovskites also impede the carriers transport. Moreover, for lead‐free halide perovskites with large bandgap or high energy emission, the poor charge injection originated from the energy‐level mismatch is a critical reason for insufficient EL characteristics. The charge injection balance needs appropriate energy band alignment and proper mobility. The energy barrier between the charge transport layers and perovskites directly determines the charge injection efficiency of LEDs. Therefore, it is crucial to choose the suitable ETLs and HTLs. We believe that exploiting multifunctional charge transporting layer (CTL) materials is also vital, wherein the CTLs can improve the charge transport and influence the heterointerfaces. Excellent multifunctional CTLs may have some active influences, such as phase control, defect passivation, ion migration reduction, and light outcoupling modulation. Some effective methods of improving EQE have been explored, which can give us some inspiration to develop LEDs based on perovskites and these methods have obtain superior results in OLEDs and QLEDs.^[^ ^226^ ^]^ For instance, out‐coupling is regarded as an efficient strategy to maximize the EQE in OLEDs and GaN LEDs. Technically, exploiting the localized surface plasmon resonance and enhancing photon extraction via a half‐ball lens and other ways have been confirmed to be effective in perovskite LEDs.^[^ ^12^, ^227^ ^]^ In addition to the LEDs, light‐emitting electrochemical cells (LECs) also show great promise as electroluminescent devices for display and lighting. Compared with LEDs, LECs are considered the simplest thin‐film lighting devices to date. Hence, developing LECs based on lead‐free halide perovskites for displays and lighting also deserves attention. To fulfill the standard of Rec.2020 defined the display color gamut, and achieve ultrahigh definition displays, laser displays based on halide perovskites are considered to be a crucial technology for the development of next‐generation display.^[^ ^136a^ ^]^ In the future, laser displays based on lead‐free halide perovskites are promising, requiring more attention in both materials and technology. In conclusion, developing lead‐free halide perovskites and fabricating lead‐free halide perovskite LEDs with outstanding properties are urgent and necessary but challenging, which requires more efforts.

## Conflict of Interest

The authors declare no conflict of interest.
